# Pregnancy complications and autoimmune diseases in women: systematic review and meta-analysis

**DOI:** 10.1186/s12916-024-03550-5

**Published:** 2024-08-26

**Authors:** Megha Singh, Fathima Fazla Ahamed Fayaz, Jingya Wang, Steven Wambua, Anuradha Subramanian, John A. Reynolds, Krishnarajah Nirantharakumar, Francesca Crowe, on behalf of MuM-PreDiCT

**Affiliations:** 1 Institute of Applied Health Research, University of Birmingham, Birmingham, UK; 2 Institute of Inflammation and Ageing, University of Birmingham, Birmingham, UK

**Keywords:** Pregnancy complications, Autoimmune disease, Pregnancy

## Abstract

**Background:**

Pregnancy complications might lead to the development of autoimmune diseases in women. This review aims to summarise studies evaluating the association between pregnancy complications and the development of autoimmune diseases in women.

**Methods:**

Medline, CINAHL, and Cochrane databases were searched up to January 2024. Nineteen pregnancy complications and 15 autoimmune conditions were included. Title, abstract, full-text screening, data extraction, and quality assessment were performed by two reviewers independently. Data were synthesised using narrative and quantitative methods. Results were presented using odds ratios (OR), relative risks (RR), incidence rate ratios (IRR), and 95% confidence intervals (CI).

**Results:**

Thirty studies were included. One study reported composite exposure to pregnancy complications had a risk of any autoimmune disease RR 3.20 (2.90–3.51) compared to women without pregnancy complications. Women with hyperemesis gravidarum had a higher risk of developing coeliac disease (*n* = 1) IRR 1.98 (1.27–2.94), Crohn’s disease (*n* = 1) IRR 1.61 (1.25–2.04), psoriasis (*n* = 1) IRR 1.33 (1.01–1.71), and rheumatoid arthritis (*n* = 2) IRR 1.35 (1.09–1.64). Miscarriage associated with subsequent diagnosis of Sjogren syndrome (*n* = 2) IRR 1.33 (1.06–2.81) and rheumatoid arthritis (*n* = 4) OR 1.11 (1.04–1.20). Gestational hypertension/preeclampsia was linked with the development of systemic sclerosis (*n* = 2) IRR 2.60 (1.10–4.60) and T1DM (*n* = 2) IRR 2.37 (2.09–2.68). Stillbirth associated with composite autoimmune conditions (*n* = 2) RR 5.82 (95% CI 4.87–6.81) and aIRR 1.25 (1.12–1.40). Postpartum psychosis was associated with autoimmune thyroid disease (*n* = 1) aIRR2.26 (1.61–2.90).

**Conclusions:**

Women with pregnancy complications subsequently had a higher risk of being diagnosed with autoimmune conditions. Whether this is due to pre-existing undiagnosed health conditions or being causally linked to pregnancy complications is not known.

**Supplementary Information:**

The online version contains supplementary material available at 10.1186/s12916-024-03550-5.

## What is already known about this subject?

- The prevalence of autoimmune conditions and pregnancy complications has increased globally.
- Women with pregnancy complications are at higher risk of cardiometabolic conditions in later life.

## What does this study add?

- This systematic review consolidates evidence from studies which have studied the association of pregnancy complications and the later development of autoimmune diseases in women.
- This review provides new knowledge to help establish the association of pregnancy complications and autoimmune diseases and identifies the need for further research to establish the true association between few conditions like the development of SLE or rheumatoid arthritis followed by miscarriage.

## How might this impact on clinical practice?

- This study will be useful for health professionals and policymakers to navigate the research findings and identify the need for clinical guidelines beyond postnatal care for women with pregnancy complications.

## Background

The prevalence of autoimmune diseases has been increasing globally over the last decade [1], and in the UK, 1 in 10 people have an autoimmune disease [1–3]. The majority of autoimmune diseases are more common in women than men [4] and are a leading cause of death in women between the age of 65 and 75 in the US and UK [5, 6]. Although the aetiology of autoimmunity is still not fully understood, the increased prevalence of autoimmune disease has been linked to defective X chromosome inactivation [7, 8] and the effects of female hormones [9].

During pregnancy, there are significant fluctuations in hormone levels and increased physiological stress. Women with pre-existing autoimmune diseases may experience flare-ups or a decrease in their symptoms. For example, rheumatoid arthritis, Grave’s disease, or psoriasis may improve during pregnancy [10–12], whilst patients with systemic lupus erythematosus (SLE) or multiple sclerosis are at an increased risk of disease exacerbations [13, 14]. With an increasing trend in pregnancy complications due to factors such as older age at pregnancy and women entering pregnancy with pre-existing long-term health conditions [15–23], it is important to study the role of pregnancy complications in the development of autoimmune diseases. Whilst it is well-established that women with autoimmune diseases have an increased risk of fertility problems and adverse pregnancy outcomes such as miscarriage and foetal growth restriction [24–30], less is known about the risk of developing autoimmunity in women who experience pregnancy complications [31]. Some studies have shown an association between parity and increased risk of Hashimoto thyroiditis, Sjögren’s syndrome, Graves’ disease, and rheumatoid arthritis [32, 33]. Moreover, the association between gestational diabetes mellitus (GDM) and the development of type 1 diabetes (T1DM) is well established [34]. Some pregnancy complications such as hyperemesis gravidarum and gestational hypertension have been associated with the development of rheumatoid arthritis [35, 36], whilst other studies have reported that pregnancy loss and gestational hypertension are associated with the development of SLE and systemic sclerosis [37, 38]. But other studies conducted on these associations have reported contradictory/inconsistent findings [39, 40].

This systematic review aims to determine the association between a wide range of pregnancy complications and the development of autoimmune diseases in women.

## Methods

This systematic review and meta-analysis have been conducted and reported according to the Preferred Reporting Items for Systematic Reviews and Meta-analyses (PRISMA) and Reporting Guidelines for Meta-analyses of Observational Studies (MOOSE) (Additional file 1 table 2 and Additional file 2 table 1) [41]. The protocol for this review was registered with Prospero CRD42023412549.

### Inclusion and exclusion criteria

Cohort, cross-sectional, or case-control studies reporting on the associations between pregnancy complications and the risk of autoimmune diseases were included. No language restrictions were applied. The population considered were pregnant women without any age restriction. The pregnancy complications (19) and autoimmune diseases (15) selected for inclusion were those that were more common in women, after consultation with experts in the subject (obstetricians, obstetric physicians, rheumatologists, and epidemiologists), and after input from patient and public involvement and engagement (PPIE) group members.

The pregnancy complications included are listed in Table 1 and autoimmune disease in Table 2.

**Table 1:** List of pregnancy complications (exposures) included in the systematic review

1. Hyperemesis Gravidarum
2. Miscarriage—missed abortion, recurrent miscarriage, spontaneous pregnancy loss or induced abortion
3. Ectopic pregnancy
4. Molar pregnancy/choriocarcinoma
5. Hypertensive disorders of pregnancy, pre-eclampsia—early or late onset, recurrent pre-eclampsia, eclampsia, HELLP (haemolysis, elevated liver enzymes, and low platelets)
6. Gestational diabetes mellitus
7. Intrahepatic cholestasis of pregnancy
8. Pelvic girdle pain
9. Placental disorders—placenta previa, placental abruption, placenta accrete, placenta percreta
10. Gestational diabetes mellitus
11. Intra-uterine growth restriction
12. Obstetric haemorrhage (postpartum)
13. Perineal trauma—third and fourth degree
14. Caesarean birth, instrumental birth
15. Postpartum depression
16. Puerperal psychosis
17. Pre-term birth, recurrent pre-term birth
18. Low birth weight
19. Small for gestational age

**Table 2:** List of autoimmune diseases (outcomes) included in the systematic review

1. Alopecia areata
2. Ankylosing spondylitis or axial spondyloarthropathy.
3. Autoimmune thyroid disease (Grave’s disease, Hashimoto’s disease)
4. Coeliac disease
5. Inflammatory bowel disease (Crohn’s disease and ulcerative colitis)
6. Multiple sclerosis
7. Myasthenia gravis
8. Psoriasis
9. Psoriatic arthritis
10. Rheumatoid arthritis
11. Sjögren’s syndrome
12. Systemic lupus erythematosus (SLE)
13. Systemic sclerosis
14. T1DM
15. Vitiligo

### Search strategy

Medline, CINAHL, and Cochrane Library were searched for studies from 2010 till January 2024.

The search strategies used pre-defined keywords of the exposures (pregnancy complications) and outcomes (autoimmune disease). Terms/keywords for each of the pregnancy complications (early adj3 pregnancy loss*, mp.miscarriage.mp, GDM) and autoimmune diseases (for example arthritis, rheumatoid systemic lupus, or SLE) were used in the search strategy. Google Scholar was searched to identify other grey literature. In addition, the reference list of the included studies, systematic reviews, and scoping reviews were searched manually to minimise the possibility of missing any relevant studies. Letters, commentaries, or editorials were excluded and studies that did not involve humans were also excluded. The searches were repeated periodically to identify newly published studies. The detailed search strategy for Medline is presented in the Additional file 1 table 1 (Table 1). This search strategy was adapted for use in other databases (CINAHL and Cochrane library). Pragmatic approach was taken given there was a substantial number of studies that needed screening (*n* = 24,340) and the study period was limited from 2010 to 2023 (*n* = 13,234). But this was complimented by a secondary search strategy looking at references of included studies, systematic reviews, scoping reviews, and by discussing with topic experts (KN, FC) to minimise the possibility of missing any study before 2010.

### Study selection

EndNote reference manager [42] was used for the title and abstract screening by two researchers independently (MS, JW). Full text of the eligible reviews was screened by two researchers independently (MS, FF). Covidence software [43] was used for full-text screening and data extraction. A third senior researcher was consulted to resolve any discrepancies in the selection of the studies (FC, KN).

### Data extraction

Two reviewers extracted data from the included studies. The data extraction form was adapted from JBI (Joanna Briggs Institute) data extraction form [44]. A standardised data extraction form was used and was piloted before use. The data were extracted for the following fields: author/s, year of publication, geographical area, aim of the study, population, exposures, comparator, outcomes, covariates, study design, definition of exposure, risk of bias assessment tool and result, number of participants included in the study, summary estimates, authors’ conclusion, and study limitations. The data extraction form is enclosed in Additional file Table 6.

### Quality assessment

The quality of included cohort, cross-sectional, and case-control studies was assessed using the Newcastle–Ottawa scale that measures study quality based on selection, comparability of the exposure and comparator groups, and the ascertainment of outcomes and exposures [45]. The scale has an overall score of 8 points for cohort or case–control studies, and 7 for cross-sectional studies with a maximum of 1 point for each numbered item within the selection and outcome/exposure categories and a maximum of 2 points for the comparability category. We defined studies with a score of ≥ 7 points as low-risk of bias studies (very good), studies with a score of 6 points as moderate-risk of bias studies (good), and those with a score of ≤ 5 points as high-risk of bias studies (satisfactory).

### Data synthesis

The effect estimates were reported as adjusted incidence rate ratios (aIRR), adjusted hazards ratios (aHR), adjusted odds ratios (aOR), or adjusted relative risks (aRR) and 95% confidence intervals (CI). We converted these effect estimates using appropriate methods (where possible) to maintain uniformity across studies [46]. Where more than one study reported the same exposure and outcome, a meta-analysis was conducted using a random effects model to generate a summary estimate. Statistical heterogeneity was estimated using the *I*^2^ statistic. To deal with potentially missing data (sample size, number exposed and unexposed), Additional file 1 of each included study was checked thoroughly, and the authors of the studies were contacted to request the data. If the data was not available and a meta-analysis could not be conducted, then effect estimates were reported as they were published. Where statistical pooling was not possible, the findings were presented in a narrative form including tables and figures to aid data presentation. R (3.3.0) and R Studio (12.1) were used to conduct statistical analysis [47–49].

### Patient and public involvement

Patient and public involvement and engagement (PPIE) representatives (RP and NM) participated in formulating the research question. They have also played key role in collaboration with clinicians and researchers to identify and consider the list of pregnancy complications and autoimmune diseases in the study. They will play a key role in disseminating the results.

## Results

**Fig. 1:**
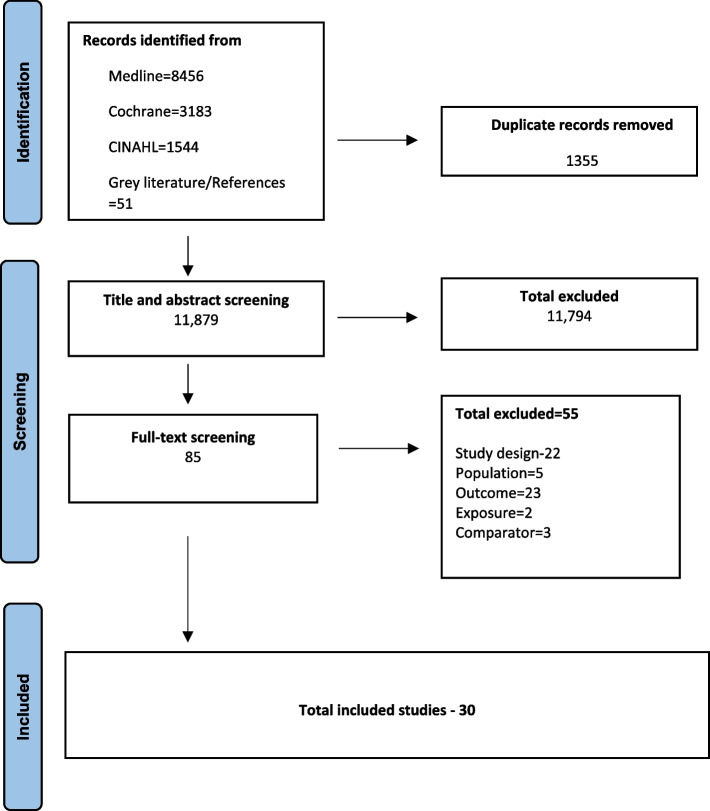
Preferred reporting items for systematic review and meta-analysis (PRISMA) flow diagram

Out of the 13,234 records identified from the search and after full-text screening of 85 studies, 30 studies were included [31, 34–36, 39, 40, 50–73]. Studies were excluded if they did not qualify in the study inclusion criteria based on the study design (*n* = 22), population (*n* = 5), intervention (2), outcome (23), and comparator(3). The selection process is shown in the PRISMA diagram (Fig. 1) [41]. The list of excluded studies is provided in Additional file 1 table 3.

Characteristics of the included studies are reported in Table 3. Out of the 30 studies, the majority were prospective cohort studies (*n* = 21), 8 were retrospective case–control studies, and 1 was a cross-sectional study. There were 23 studies conducted in Europe, the remainder were in Taiwan (2), China (2), South Korea (1), and the United States (2). In most cohort studies (*n* = 14), information about the pregnancy complications and autoimmune diseases was collected through medical records. Medical records were used to establish the autoimmune diseases in case–control studies, and questionnaires were used to determine the pregnancy complications. The follow-up period of the cohort studies varied from 9 months to 26 years with a median of 12 years. Out of the 21 cohort studies, 9 used the data from the same cohort (Danish national registry) [31, 36, 39, 53, 54, 63, 64, 69]. In the instance of two studies reporting the same exposure and outcome, the most recent study was used to avoid duplication. We have done this in accordance with the Cochrane handbook for systematic reviews [74]. For instance, two studies, Mikkelsen et al. [63] and Nielsen et al. [64], were both reporting the association of pregnancy complications and the future development of multiple sclerosis in women using Danish Civil Registration System. Mikkelsen et al.’s study was used to report the findings in this review. Details have been added in Additional file tables 1 and 5 [75, 76]. A total of 18 different pregnancy complications and 12 autoimmune diseases (including an overall “all autoimmune diseases”) were investigated across the studies. Rheumatoid arthritis (8 studies) and SLE (5 studies) were two of the most included outcomes. The meta-analysis performed in this systematic review is included in Additional file 1 figure 1.1–1.11.

**Table 3:** Basic characteristics of included thirty studies

**Serial number**	**Author, year**	**Geographical area**	**Database/heath care setting**	**Sample size**	**Pregnancy complication(s) considered (exposure)**	**Autoimmune disease(s) considered (outcome)**
**Prospective cohort studies**						
1.	Auvinen 2020 [34]	Finland	University hospital	782	Gestational diabetes mellitus	Type 1 diabetes mellitus
2.	Bergink, 2011 [50]	The Netherlands	Department of psychiatry at the Erasmus Medical Centre for evidence of postpartum psychosis	148	Postpartum psychosis	Autoimmune thyroid dysfunction
3.	Bergink 2018 [51]	Denmark	Danish national registry	312,779	Postpartum psychosis	Autoimmune thyroid dysfunction
4.	Bränn 2023 [73]	Sweden	Swedish National Medical Birth Register	530,397	Perinatal depression	Autoimmune diseases
5.	Hardy 1999 [52]	UK	Patients from Nottingham	314	Pregnancy loss (spontaneous abortion, stillbirth, miscarriage, or ectopic pregnancy), Induced abortion	Systemic lupus erythematosus
6.	Harpsoe 2013 [53]	Denmark	Danish National Birth Cohort	56,108	Hyperemesis gravidarum, gestational hypertension, pre-eclampsia	Inflammatory bowel disease
7.	Jørgensen 2010 [36]	Denmark	Danish civil Registration System	7017	Hyperemesis gravidarum, ectopic pregnancies, miscarriage, Induced abortions missed abortions, hydatidiform moles, gestational hypertension, and pre-eclampsia	Rheumatoid arthritis
8.	Jørgensen 2012 [54]	Denmark	Danish women born between 1955 and 1993	1,564,567	Hyperemesis gravidarum, gestational hypertension, preeclampsia, spontaneous abortions, missed abortions, ectopic pregnancies	Autoimmune diseases
9.	Jørgensen 2014 [39]	Denmark	Danish National Birth Cohort	97,077	Gestational hypertension, pre-eclampsia, hyperemesis gravidarum	Rheumatoid arthritis
10.	Kamper 2018 [56]	Denmark	Danish Medical Birth Register	778, 758	Pre-eclampsia	Systemic sclerosis
11.	Khashan 2011 [31]	Denmark	Danish Civil Registration System, the Danish National Hospital Register [28] and the Danish Medical Birth Register	1,035,639	Caesarean section, induced abortion	Any autoimmune disease
12.	Lee 2022 [59]	South Korea	National health insurance corporation	2,260,952	Caesarean section	Systemic sclerosis
13.	Lin 2018 [60]	Taiwan	National Health Insurance Research Database	45,451	Postpartum depression	Autoimmune diseases
14.	Lin 2016 [61]	Taiwan	National Health Insurance programme	145,455	Gestational hypertension	Systemic lupus erythematosus
15.	Mao 2022 [62]	China	National Health and Nutrition Examination Survey cohort	11,997	Gestational diabetes mellitus	Rheumatoid arthritis
16.	Mikkelsen 2022 [63]	Denmark	Danish Civil Registration System	1,513,560	Miscarriage	Myasthenia gravis
17.	Nielsen 2011 [64]	Denmark	Danish Civil Registration System	6332	Miscarriage, ectopic pregnancy, hyperemesis gravidarum, gestational hypertension, pre-eclampsia, stillbirth,	Multiple sclerosis
18.	Savitz 2014 [65]	USA	Data on all births in hospitals in New York City, New York	849,639	Gestational hypertension	Type 1 diabetes mellitus
19.	Stuart 2018 [68]	Sweden	Swedish National Board of Health and the data	1,873,440	Low birth weight	Type 1 diabetes mellitus
20.	Ulff-Møller 2009 [69]	Denmark	Civil Registration System	2,140,000	Abortion (spontaneous, missed, induced), ectopic pregnancy, molar pregnancy	Systemic lupus erythematosus
21.	Wikstrom 2015 [72]	Sweden	Swedish Medical Birth Register	125,281	Intrahepatic cholestasis of pregnancy	Inflammatory bowel disease
**Retrospective case–control studies**						
1.	Julkunen 1993 [55]	Finland	Fourth and Second Departments of Medicine and the Department of Dermatology of the Helsinki University Central Hospital	659	Spontaneous abortion, stillbirth, preterm birth, intrauterine growth restriction	Systemic lupus erythematosus
2.	Kay 1965 [57]	UK	American Rheumatism Association	418	miscarriage, stillbirth	Rheumatoid arthritis
3.	Kither 2020 [58]	UK	Clinical Practice Research Datalink	117,446	miscarriage, gestational hypertension, intrauterine growth restriction, placental complications Stillbirth	Connective tissue disease, Systemic lupus erythematosus
4.	Ma 2014 [40]	USA	Group Health Cooperative, a large Seattle-based consumer-governed non-profit health care system	1304	Low birth weight, small for gestational age	Rheumatoid arthritis
5.	Siamopoulou 1988 [66]	Greece	Greek married women	573	miscarriage, preterm birth, stillbirth,	Systemic lupus erythematosus, Rheumatoid arthritis
6.	Spector 1990 [67]	UK	American Rheumatism Association criteria	657	miscarriage induced abortion, stillbirth	Rheumatoid arthritis
7.	Van Wyk 2011 [70]	The Netherlands	Leiden University Medical Centre (LUMC) and the VU Medical Centre of Amsterdam (VUMC) in the Netherlands	206	miscarriage, gestational hypertension, intrauterine growth restriction	Systemic sclerosis
8.	Wallenius 2011 [71]	Norway	Medical Birth Registry of Norway	335,377	Pre-eclampsia, caesarean section, postpartum haemorrhage, low birth weight, preterm delivery, small for gestational age,	Chronic inflammatory arthritis RA/PSA/ANK S
**Cross-sectional study**						
1.	Hee 2022 [35]	China	China Kadoorie Biobank (CKB)	302,510	Miscarriage	Rheumatic arthritis

*NOS* Newcastle–Ottawa’s scale for quality assessment of the cohort, cross-sectional, or case–control studies, *RA/PSA/ANK S* Rheumatoid arthritis, psoriatic arthritis, and ankylosing spondylitis, *T1DM* type 1 diabetes mellitus

### Quality assessment

**Fig. 2:**
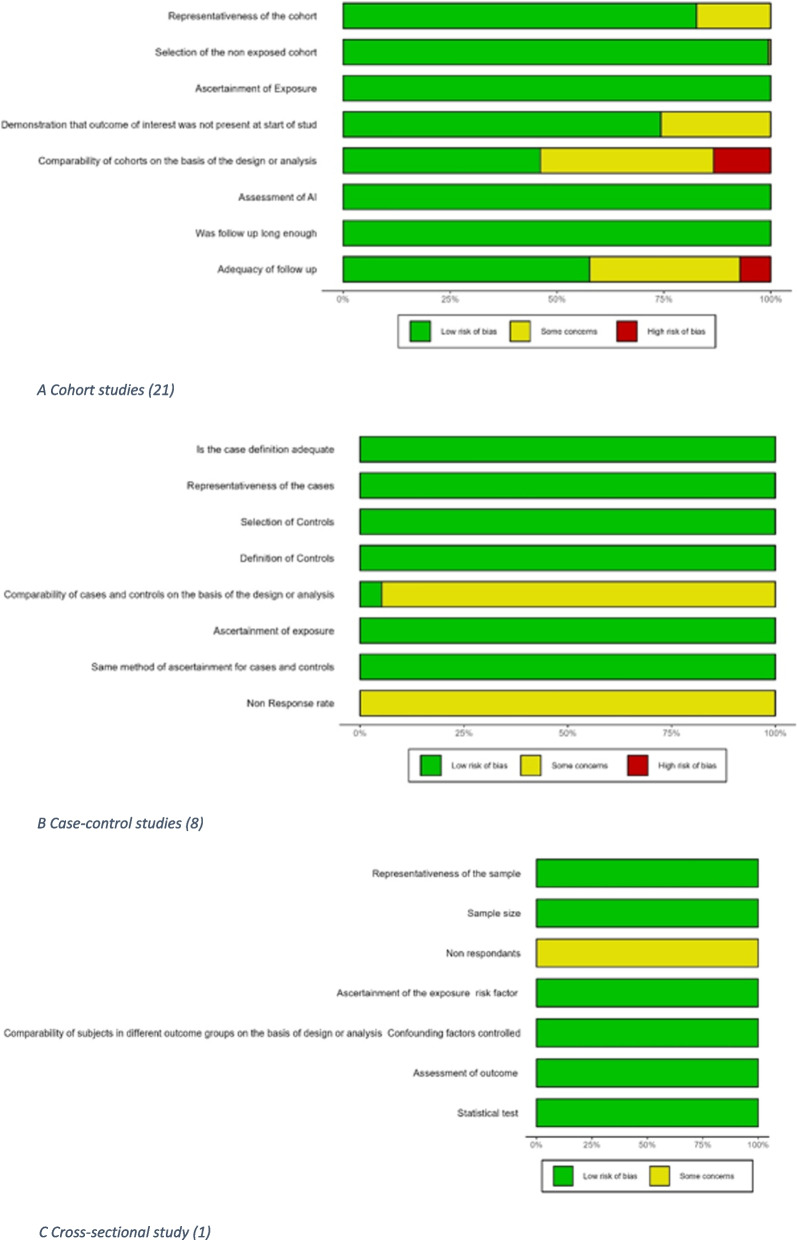
Quality assessment of included studies (Newcastle–Ottawa scale). **A** Cohort studies (21). **B** Case–control studies (8). **C** Cross-sectional study (1)

Results of the quality assessment of the studies using the Newcastle–Ottawa scale are shown in Fig. 2 and Additional file 1 Table 4.1–4.3 [77]. Eighteen out of 30 studies had a low risk of bias with an overall “very good” rating. The principal areas of concern were the comparability of cohorts and the adequacy of follow-up.

### All autoimmune diseases

There was more than a threefold higher risk of developing autoimmune diseases (*n* = 7) in women with pregnancy complications (*n* = 6) RR 3.20 (95% CI 2.90–3.51) when compared with women without pregnancy complications [58]. Out of the pregnancy complications studied individually, two studies reported, women with previous miscarriage RR 3.41 (3.03–3.85) and aIRR 1.10 (1.07–1.14) was reported to have higher risk [58]. One cohort and one case–control study reported a higher risk of autoimmune diseases in women with gestational hypertension or pre-eclampsia; RR 2.05 (1.70–2.48) and aIRR 1.21 (1.16–1.26) [54, 58], respectively. Women with stillbirth were reported to have higher chances to have autoimmune disease in later life reported by two studies RR 5.82 (4.97–6.81) [58] and aIRR 1.25 (1.12–1.40) [54]. There was a significantly higher risk of developing autoimmune diseases for women with preterm birth RR 2.35 (1.89–2.92) [58]. There was little association reported with caesarean section, induced abortion, or postpartum depression with the development of autoimmune diseases [31, 54, 61]. However, a study reported a higher risk of developing autoimmune diseases in women with perinatal depression aHR 1.52 (1.46–1.58), with antenatal depression aHR 1.50 (1.43–1.58), and postpartum depression aHR 1.55 (1.45–1.65) [73].

### Autoimmune thyroid diseases

Hyperemesis gravidarum aIRR 1.49 (1.28–1.72), gestational hypertension or pre-eclampsia aIRR 1.20 (1.10–1.30), and postpartum depression aHR 1.57 (1.05–2.33) [54, 60] were all associated with a higher risk of Grave’s disease but there was no significant association between ectopic pregnancy and Grave’s disease aIRR 1.04 (0.93–1.17) [54]. Gestational hypertension/pre-eclampsia aIRR 1.41 (1.17–1.68) was associated with a higher risk of Hashimoto’s thyroiditis but there was little association of hyperemesis gravidarum or ectopic pregnancy with Hashimoto’s thyroiditis; aIRR 1.38 (0.95–1.92) and aIRR 0.92 (0.68–1.21), respectively [54]. Two cohort studies reported a greater risk of autoimmune thyroid disease in women with postpartum psychosis aOR 2.78 (1.08–7.17) at 9 months and aIRR 2.26 (1.61–2.90) with 2 years follow-up postpartum when compared with women without postpartum psychosis [50, 51].

### Coeliac disease

Women who experienced hyperemesis gravidarum had almost a twofold risk of coeliac disease compared to women without; aIRR 1.98 (1.27–2.94) [54]. None of the other pregnancy complications were significantly associated with coeliac disease; ectopic pregnancy aIRR 1.12 (0.75–1.61), gestational hypertension, pre-eclampsia aIRR 1.19 (0.89–1.56), and intrahepatic cholestasis of pregnancy aHR 1.20 (0.82–1.74) [54, 72].

### Inflammatory bowel disease (Crohn’s disease and ulcerative colitis)

Out of the five pregnancy complications reported for IBD (hyperemesis gravidarum, missed abortion, gestational hypertension, pre-eclampsia, and Caesarean section), none of these associations were statistically significant. However, studies reporting ulcerative colitis and Crohn’s disease separately found significant associations. Hyperemesis was significantly associated with the development of both ulcerative colitis and Crohn’s disease, aIRR 1.34 (1.09–1.62) and aIRR 1.61 (1.25–2.04), respectively [54]. Furthermore, a higher risk of Crohn’s disease was also observed in women with intrahepatic cholestasis of pregnancy, HR 1.55 (1.14–2.10) [72]. No other pregnancy complications were associated with the development of IBD as reported in Fig. 4.

### Ankylosing spondylitis

Out of the three pregnancy complications studied with the development of ankylosing spondylitis in women, there was no significant association for hyperemesis gravidarum IRR 1.63 (0.96–2.25) or ectopic pregnancy IRR 1.02 (0.66–1.50); a significant association was noted with gestational hypertension and pre-eclampsia IRR 1.40 (1.06–1.82) [54] (Fig. 5).

### Rheumatoid arthritis

Out of the five studies reporting the association of miscarriage and rheumatoid arthritis, four were meta-analysed to estimate 11% higher odds: pooled OR 1.11 (1.04–1.20) with the other study showing a slightly elevated risk that was not statistically significant aIRR 1.06 (0.97–1.15) [35, 36, 54, 57, 66, 67]. A significant association was also reported with hyperemesis, gestational hypertension, and pre-eclampsia with aIRR 1.35 (1.09–1.64), aIRR 1.18 (1.05–1.31), respectively [54]. [35, 36, 54, 57, 66, 67]. Whilst three studies were pooled to derive a significant association between rheumatoid arthritis and induced abortion 1.46 OR (1.01–2.12). Women with any pregnancy loss were reported to be at higher risk of developing the disease in one study aIRR 1.12 (1.06–1.12) and others reported no association aIRR 1.01 (0.67–1.44), and this will require further research to establish the true association. The association for induced abortion or any pregnancy loss with rheumatoid arthritis reported mixed findings with significant association reported by few studies and insignificant by others as shown in Fig. 5 [31, 35, 36, 54, 57, 66, 67]. A higher risk of developing rheumatoid arthritis was observed in women who delivered “extremely low birth weight” babies (< 1000 g) with RR 3.70 (1.00–13.20) or “low birth weight” babies (< 2500 g) with RR 1.40 (1.00–2.10) when compared to women who delivered normal birth weight babies [40]. An increased risk of rheumatoid arthritis was also reported for women with postpartum depression with aHR 2.62 (1.28–5.39) [60]. No other pregnancy complications studied in relation with the development of rheumatoid arthritis were statistically significant (Fig. 5). There was also no significant association with the development of rheumatoid arthritis as reported in women who delivered very low birth weight babies (< 1500 g) in a study with a small sample size (*n* = 20) [40].

### Rheumatoid arthritis, psoriatic arthritis, and ankylosing spondylitis (composite outcome)

There were no other significant associations with rheumatoid arthritis or the composite outcome with other pregnancy complications studied (gestational hypertension or pre-eclampsia, caesarean section, postpartum haemorrhage, or mothers delivering preterm births or low birth weight babies) (Fig. 5) [71]. Women who delivered small for gestational age babies were more likely to have the composite outcome of rheumatoid arthritis, psoriatic arthritis, and ankylosing spondylitis aOR 1.60 (1.00–2.56) when compared to women with normal for gestational age babies.

### Connective tissue diseases (systemic lupus erythematosus, Sjogren syndrome, systemic sclerosis)

#### Connective tissue disease

Seven pregnancy complications were reported in association with connective tissue disease (CTD). Women with placental abruption or preterm birth had significantly higher risk of CTD; RR 3.39 (1.96–5.89) and RR 1.78 (1.12–2.82), respectively, [58] in Fig. 6. The associations of gestational hypertension, intrauterine growth restriction (IUGR), miscarriage, stillbirth, and composite pregnancy complications with CTD were not statistically significant [58].

#### Systemic lupus erythematosus (SLE)

Results for an association between miscarriage and SLE were mixed with one study reporting aIRR of 1.43 (1.08–1.88), and a pooled RR for two smaller studies showing no significant association 0.94 (0.37–2.40) [54, 58, 66, 69]. However, there was a significant association reported with any pregnancy loss and future development of the disease with OR 1.87 (1.31–2.67) [52, 55]. Missed abortions were associated with a higher risk of SLE; IRR 2.13 (1.48–2.98) [54, 69]. Women with history of IUGR had almost a fivefold higher risk of SLE; RR 4.80 (1.60–14.50) when compared with women with no IUGR [55]. Results from three studies showed that a history of stillbirth was associated with a four times higher risk of SLE, pooled RR 4.01 (3.11–5.17) and pooled IRR 3.29 (3.22–4.88) [54, 58, 66, 69].

#### Systemic sclerosis

There was an association between gestational hypertension or pre-eclampsia and systemic sclerosis in two studies: OR 2.60 (1.10–4.60). Kamper et al. [56] reported a significant association with the development of localised scleroderma in women with pre-eclampsia with IRR 1.69 (1.02–2.80) but a nonsignificant association with subset of systemic disease aIRR 1.46 (0.75–2.80) [56, 70]. There was a three- to fourfold higher risk of systemic sclerosis for women with IUGR compared with women with normal foetal growth OR 3.90 (1.20–12.30) [70] and caesarean birth compared to vaginal birth RR 3.09 (1.96–4.63) [59].

#### Sjögren’s syndrome

There were seven pregnancy complications examined in relation to Sjögren’s syndrome with results showing a greater risk with hyperemesis gravidarum, aIRR 1.79 (1.06–2.81); miscarriage, aIRR 1.33 (1.08–1.63); induced abortion, aIRR 1.18 (1.01–1.38); and gestational hypertension or pre-eclampsia, aIRR 1.43 (1.09–1.85) [54]. The associations for ectopic pregnancy, missed abortion, or preterm birth, aIRR 1.18 (0.79–1.68), aIRR 1.12 (0.80–1.51), and RR 0.09 (0.04–18.09), respectively [54, 58, 66], were not statistically significant.

#### Type 1 diabetes mellitus (T1DM)

Hyperemesis gravidarum aIRR 1.05 (0.74–1.45) and ectopic pregnancy aIRR 1.06 (0.58–1.77) were not significantly associated with T1DM [34, 54, 65, 68] in Fig. 7. Results from one cohort study showed that gestational hypertension or pre-eclampsia was associated with a twofold higher risk of T1DM; aIRR 2.37 (2.09–2.68) [68], whereas in the other study, the association was higher but not statistically significant; OR 1.80 (0.80–3.80) [65]. Results from two studies showed that the risk of T1DM for women with gestational diabetes was considerably higher; pooled OR 40.89 (24.31–68.78) [34, 65]. There was almost a fourfold higher risk of T1DM in women who delivered large for gestational age babies compared to women delivering normal weight for gestational age babies aHR 3.60 (3.23–4.01) [68]. There was no significant association for women who delivered small for gestational age babies and T1DM; aHR 1.11 (0.94–1.30) [62, 71].

#### Psoriasis

Out of the seven pregnancy complications, five complications were associated with a higher risk of psoriasis: hyperemesis gravidarum HR 1.33 (1.01–1.71), ectopic pregnancy aIRR 1.28 (1.07–1.53), induced abortions aIRR 1.33 (1.24–1.42), gestational hypertension or pre-eclampsia aIRR 1.22 (1.06–1.40), and intrahepatic cholestasis of pregnancy aIRR 1.27 (1.07–1.51) [54, 61, 72]. Missed abortion and postpartum depression were not significantly associated with the risk of psoriasis (Fig. 7) [54, 61].

#### Associations of pregnancy complications with other miscellaneous autoimmune conditions

No significant association was reported with pregnancy complications studied (hyperemesis, ectopic pregnancy, miscarriage, or gestational hypertension) and the development of multiple sclerosis as mentioned in Fig. 7 [64]. The association of postpartum depression and alopecia areata (HR 1.97, 0.72–5.37) [60], and recurrent pregnancy loss and myasthenia gravis (RR 0.85, 0.54–1.31) were not statistically significant [63].

#### Timing of developing autoimmune diseases following pregnancy/pregnancy complications

Four studies reported the occurrence of autoimmune diseases following pregnancy over different follow-up times [31, 51, 54, 69]. Following a pregnancy complication, a woman’s risk of developing Grave’s disease or SLE was higher in the early years after childbirth in comparison to later in life [35, 39, 54, 63, 69]. For instance, there was a higher risk of SLE in women with pregnancy loss in the first year postpartum IRR 2.64 (1.18–6.29), whereas there was no significant association noted after two or more years postpartum IRR 1.90 (0.87–4.48) [69]. Conversely, the risk of developing rheumatoid arthritis (RR 1.05; 0.98–1.13, 5+ years: RR 2.24; 1.58–3.05, and multiple sclerosis) (RR 1.00; 0.91–1.09, 5+ years: RR 2.20; 1.72–2.77) was greater after 5 or more years postpartum. Also, women with hyperemesis gravidarum were at a greater risk of developing rheumatoid arthritis in the first 4 years post birth; this reduced after 5 years, IRR 1.40 (1.09–1.76) and IRR 1.02 (0.59–1.11), respectively [54, 69]

## Discussion

**Fig. 3:**
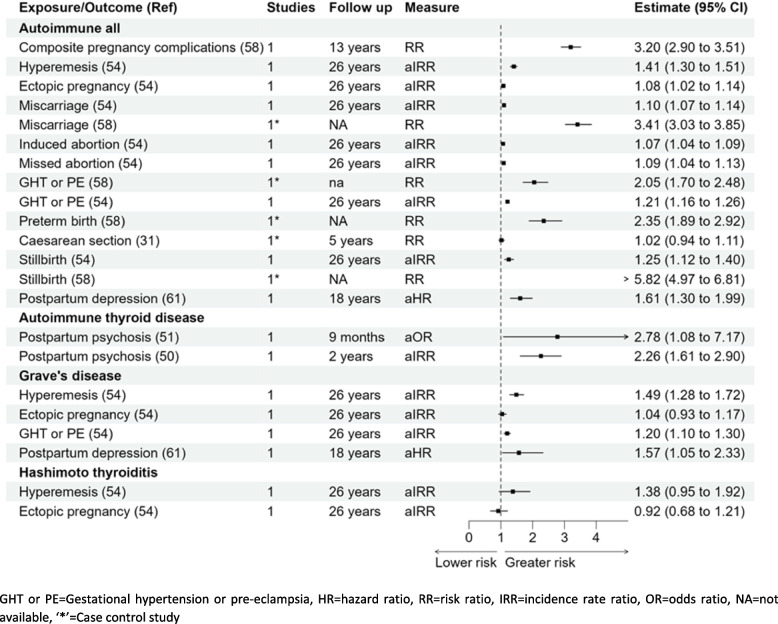
Forest plot association of pregnancy complications and autoimmune diseases (overall) and autoimmune thyroid diseases

**Fig. 4:**
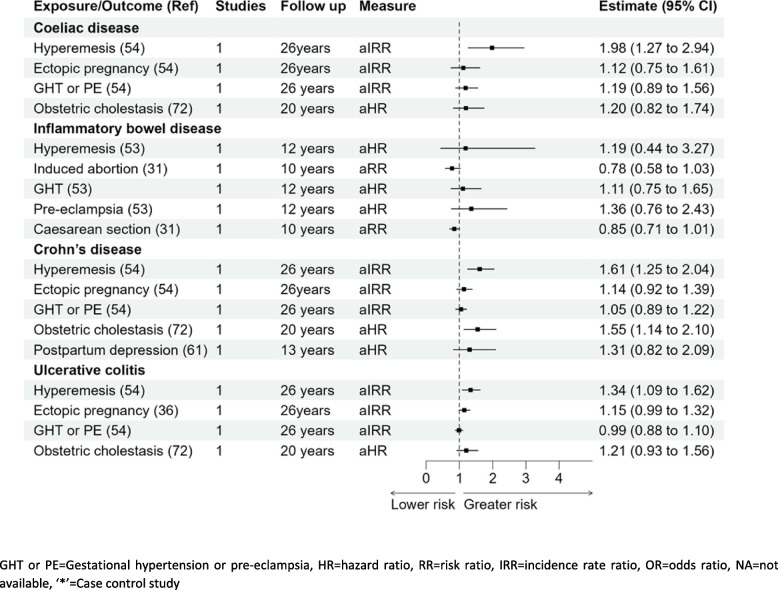
Forest plot-association of pregnancy complications and coeliac disease or inflammatory bowel disease (Crohn’s disease and ulcerative colitis)

**Fig. 5:**
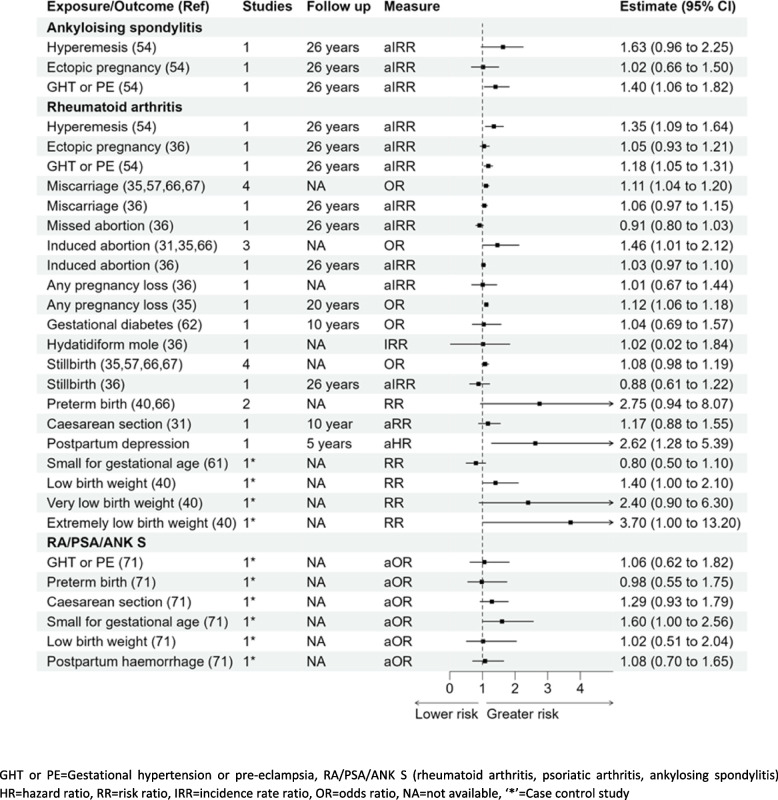
Forest plot- association of pregnancy complications and Ankylosing spondylitis, rheumatoid arthritis, or RA/PSA/ANK S

**Fig. 6:**
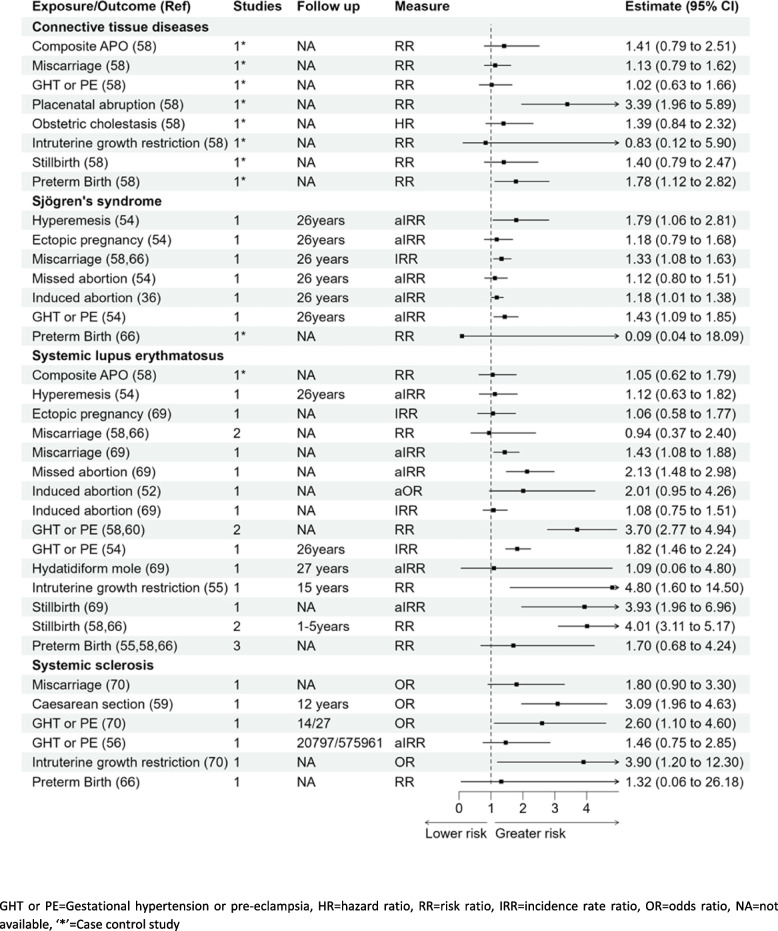
Forest plot showing the association of pregnancy complications and connective tissue diseases

**Fig. 7:**
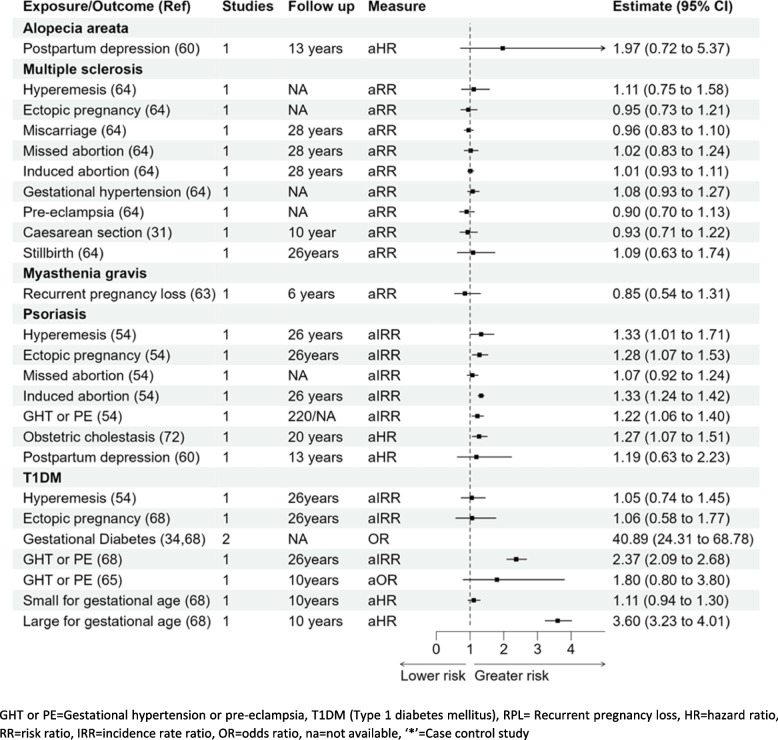
Forest plot showing the association of pregnancy complications and alopecia areata, multiple sclerosis, myasthenia gravis, psoriasis, or T1DM

This systematic review provides an overview of the associations of 18 pregnancy complications with the risk of developing 15 autoimmune diseases. This review compiles all the available evidence on pregnancy complications linked to the development of autoimmune diseases in women in later life (Figs. 2, 3, 4, 5, 6 and 7) and generates new evidence by quantitative or qualitative analysis of the studies studying the same exposure and outcomes (Additional file 1 figure 1.10). This also further points out the differences in the results observed in two or more studies analysing the association of the same pregnancy complication and autoimmune disease (Additional file 1 fig. 1.11).

Studies reported associations for gestational hypertension/preeclampsia followed by preterm birth, hyperemesis gravidarum and ectopic pregnancy, and future autoimmunity. However, there was little or no research on some complications such as molar pregnancy or placental disorders. From the perspective of autoimmune disease outcomes, most studies examined the associations with rheumatoid arthritis followed by autoimmune thyroid diseases, Sjögren’s syndrome, and psoriasis. In contrast, there were very few studies that included vitiligo or myasthenia gravis.

Many of the pregnancy complications increased the risk of overall autoimmune diseases almost threefold, particularly hyperemesis gravidarum, miscarriage, gestational hypertension, stillbirth, and antenatal/postpartum depression. Apart from the known association of gestational diabetes and T1DM, results from this review showed that IUGR or stillbirth were associated with almost three- to fourfold increased risk of systemic sclerosis or systemic lupus erythematosus. There was a higher risk of rheumatoid arthritis with preterm birth and low birth weight babies.

There are findings which require further research, for example, the association of miscarriage and development of SLE [58, 65, 69] and the association of gestational hypertension with T1DM [54, 65]. The difference in the findings could possibly be due to the varying study designs or difference in the sample size of the studies. There had been mixed findings amongst the studies included and these may be due to the varying sample size or the study designs.

Earlier studies focused on the association of pregnancy complications with child outcomes such as caesarean birth or pre-eclampsia and the association with long-term health conditions in babies [78–84]. However, more recently, studies have reported the association of pregnancy complications and the development of long-term conditions in the mother [31, 85]. Reproductive factors and pregnancy complications were found to be associated with later development of metabolic conditions [86–88]. An association between pregnancy itself, irrespective of pregnancy complications, and the development of autoimmune diseases has been reported [31]. Some studies identified the association between parity and the development of systemic sclerosis; however, the findings have been conflicting [89–92].

It is not clear whether the observed pregnancy complications occur in women with preclinical autoimmune disease or whether these events directly pre-dispose to the development of autoimmune disease [93]. In terms of the former, women with undifferentiated connective tissue disease (UCTD), who have features compatible with a CTD but do not have a defined CTD [94], have an increased risk of pregnancy complications including premature delivery, pre-eclampsia, and stillbirth [95]. As approximately 30% of UCTD may progress to CTD, typically SLE, it is possible that some of the pregnancy complications occurred in women who were in the initial stages of UCTD, i.e. the pregnancy complications were due to a subclinical autoimmune disease.

On the other hand, pregnancy/pregnancy complications bring about fluctuations in female sex hormones accompanied by physiological stress [96]. The blood levels of both oestrogen and progesterone increase rapidly from the middle of the second trimester, peaking at term. Oestrogen and progesterone have broad effects on the function of both innate and adaptive immune cells (including monocytes/macrophages, neutrophils, dendritic cells, and T and B lymphocytes) [97]. In pregnancy, placental production of oestriol (E3) increases dramatically. Oestriol has potent anti-inflammatory effects including reducing pro-inflammatory cytokine production, increasing anti-inflammatory cytokines, and reducing CD4+ and CD8+ T cells [98]. Similarly, progesterone increases regulatory T cells and reduces natural killer cell function systemically and within the placenta [98]. It is possible, therefore, that hormonal fluctuations and the loss of this anti-inflammatory state postpartum could accelerate the development of autoimmune disease. Furthermore, oestrogens reduce B cell apoptosis which, whilst contributing to maternal humoral immunity, may promote autoreactive B cell survival and drive the immune system toward autoimmunity [99].

A key driver of future autoimmune disease may be foetal microchimerism [100]. Foetal cells are present at a low frequency in the maternal circulation postpartum and may persist for decades [57, 101–103]. Foetal origin microchimerism is observed at increased rates during pregnancy complications such as miscarriage, pre-eclampsia, foetal growth restriction [104], or premature labour [102, 105]. The mechanisms by which foetal microchimeric cells mediate an increased risk of autoimmunity is not understood although an increased number of these cells is observed in the thyroid gland of women with autoimmune thyroid disease [106]. To date, a pathogenic role for foetal microchimeric cells has not been demonstrated, and these cells may induce maternal tolerance to foetal antigens and via a bystander effect reduce the severity of some autoimmune diseases such as RA during pregnancy [106].

Our study has several strengths. The scope of our review was broad and summarises the association of pregnancy complications and the subsequent development of a wide range of autoimmune diseases, and we were able to perform a meta-analysis of studies reporting the same exposure and outcome where possible. We employed rigorous methodology with a pre-specified protocol, and our systematic search was conducted without language restriction and two reviewers screened, extracted data, and appraised the quality of the studies.

There are, however, some limitations. A meta-analysis could not be performed for some of the studies due to missing data such as the sample size or number of exposed/unexposed. Some of the results reported therefore are as reported in one study. Also, nine studies were conducted using the same cohort (Danish birth cohort); this may have a disproportionate effect on our findings. However, efforts were made to avoid duplication in the reporting of results. This study is not able to determine causality and there is a possibility that the women already have undiagnosed preclinical autoimmune diseases, which increased their risk of pregnancy complications in studies, especially those with shorter follow-up time.

Additional research is required that incorporates a comprehensive analysis of pregnancy complications and characterise the phenotype and functionality of persistent foetal origin cells in women with autoimmune diseases compared with healthy women. The exact pathophysiology behind the development of these conditions remains unclear and we do not know why some pregnancy complications have a larger effect than others. To address these questions, prospective longitudinal studies following up on women who experienced pregnancy complications are needed, observing when autoantibodies are first detected [107]. Furthermore, larger epidemiological studies would be required to define whether autoimmune disease is more prevalent in women who have experienced pregnancy complications and if there is a clear underlying association.

## Conclusions

This review has reported that there is an association between pregnancy complications and the subsequent development of autoimmune diseases in women. To further address this question, prospective longitudinal studies following up on women who experienced pregnancy complications are needed, observing when autoantibodies are first detected. Meanwhile, clinicians should be vigilant and detect autoimmune conditions early in women with a history of pregnancy complications.

### Supplementary Information

Additional file 1: Table 1. Search Strategy MEDLINE. Table 2. The Preferred Reporting Items for Systematic reviews and Meta-Analyseschecklist. Table 3. List of excluded studies. Table 4.1. Quality assessment of the Cohort studies using NOS. Table 4.2. Quality assessment of the case control studies using NOS. Table 4.3. Quality assessment of the cross-sectional studies using NOS. Figure 1.1. Meta-analysis of two studies reporting association of miscarriage and future development of SLE. Figure 1.2. Meta-analysis of two studies reporting association of miscarriage and future development of rheumatoid arthritis. Figure 1.5. Meta-analysis of two studies reporting association of stillbirth and future development of SLE. Figure 1.6. Meta-analysis of two studies reporting association of gestational hypertension or pre-eclampsia and future development of SLE. Figure 1.7. Meta-analysis of two studies reporting association of preterm birth and future development of SLE. Figure 1.8. Meta-analysis of two studies reporting association of preterm birth and future development of rheumatoid arthritis. Figure 1.9. Meta-analysis of two studies reporting association of gestational diabetes and future development of T1DM. Figure 1.10. The new findings from this review. Figure 1.11. The mixed findings of this review. Table 5. Cohort studies with same or overlapping cohorts. Table 6. Data Extraction form.

Additional file 2.

## Data Availability

No datasets were generated or analysed during the current study.

## Abbreviations

95% CI: 95% Confidence intervals
aHR: Adjusted hazard ratio
aIRR: Adjusted incidence risk ratio
aOR: Adjusted odds ratio
aRR: Adjusted risk ratio
AxSpA: Axial spondyloarthropathy
CD: Clusters of differentiation
CS: Caesarean section
CTD: Connective tissue diseases
GDM: Gestational diabetes mellitus
GHT or PE: Gestational hypertension or pre-eclampsia
HELLP: Haemolysis, elevated liver enzymes, and low platelet syndrome
IBD: Inflammatory bowel disease
IUGR: Intrauterine growth retardation
LBW: Low birth weight
MESH: Medical subject headings
MOOSE: Meta-analyses of observational studies
MS: Multiple sclerosis
NA: Not applicable
NOS: Newcastle–Ottawa scale
PPIE: Patient and public involvement and engagement
PRISMA: Preferred reporting items for systematic review and meta-analysis
RA/PSA/ANK S: Rheumatoid arthritis, psoriatic arthritis, and ankylosing spondylitis
SGA: Small for gestational age
SLE: Systemic lupus erythematosus
T1DM: Type 1 diabetes mellitus
UCTD: Undifferentiated connective tissue disease
UK: United Kingdom
US: United States
