# Supplementary material for: Pregnancy complications and autoimmune diseases in women: systematic review and meta-analysis
Source: BMC Med. 2024 Aug 26;22:339. doi: 10.1186/s12916-024-03550-5 (PMC11346028; doi:10.1186/s12916-024-03550-5)
Supplement: Supplementary file 1 — Additional file 1: Table 1. Search Strategy MEDLINE. Table 2. The Preferred Reporting Items for Systematic reviews and Meta-Analyseschecklist. Table 3. List of excluded studies. Table 4.1. Quality assessment of the Cohort studies using NOS. Table 4.2. Quality assessment of the case control studies using NOS. Table 4.3. Quality assessment of the cross-sectional studies using NOS. Figure 1.1. Meta-analysis of two studies reporting association of miscarriage and future development of SLE. Figure 1.2. Meta-analysis of two studies reporting association of miscarriage and future development of rheumatoid arthritis. Figure 1.5. Meta-analysis of two studies reporting association of stillbirth and future development of SLE. Figure 1.6. Meta-analysis of two studies reporting association of gestational hypertension or pre-eclampsia and future development of SLE. Figure 1.7. Meta-analysis of two studies reporting association of preterm birth and future development of SLE. Figure 1.8. Meta-analysis of two studies reporting association of preterm birth and future development of rheumatoid arthritis. Figure 1.9. Meta-analysis of two studies reporting association of gestational diabetes and future development of T1DM. Figure 1.10. The new findings from this review. Figure 1.11. The mixed findings of this review. Table 5. Cohort studies with same or overlapping cohorts. Table 6. Data Extraction form. [file 12916_2024_3550_MOESM1_ESM.docx]

Contents

[Table 1 Search Strategy MEDLINE 2](#_Toc152749727)

[Table 2 The Preferred Reporting Items for Systematic reviews and Meta-Analyses (PRISMA) checklist 6](#_Toc152749728)

[Table 3 List of excluded studies 7](#_Toc152749729)

[Table 4.1 Quality assessment of the Cohort studies using NOS (Newcastle Ottawa scale) 12](#_Toc152749730)

[Table 4.2 Quality assessment of the case control studies using NOS (Newcastle Ottawa scale) 14](#_Toc152749731)

[Table 4.3 Quality assessment of the cross-sectionall studies using NOS (Newcastle Ottawa scale) 15](#_Toc152749732)

[Figure 1.1 Meta-analysis of two studies reporting association of miscarriage and future development of SLE 15](#_Toc152749733)

[Figure 1.2 Meta-analysis of two studies reporting association of miscarriage and future development of rheumatoid arthritis (odds ratios 16](#_Toc152749734)

[Figure 1.3 Meta-analysis of two studies reporting association of induced abortion and future development of rheumatoid arthritis 16](#_Toc152749735)

[Figure 1.4 Meta-analysis of two studies reporting association of miscarriage and future development of rheumatoid arthritis (odds ratios) 16](#_Toc152749736)

[Figure 1.5 Meta-analysis of two studies reporting association of stillbirth and future development of SLE(risk ratio) 17](#_Toc152749737)

[Figure 1.6 Meta-analysis of two studies reporting association of gestational hypertension or pre-eclampsia and future development of SLE 17](#_Toc152749738)

[Figure 1.7 Meta-analysis of two studies reporting association of preterm birth and future development of SLE 17](#_Toc152749739)

[Figure 1.8 Meta-analysis of two studies reporting association of preterm birth and future development of rheumatoid arthritis 18](#_Toc152749740)

[Figure 1.9 Meta-analysis of two studies reporting association of gestational diabetes and future development of T1DM 18](#_Toc152749741)

[Figure 1.10 Figure 1.10 The new findings from this review 23](#_Toc152749754)

[Figure 1.11 The mixed findings of this review 23](#_Toc152749755)

[Table 5 Cohort studies with same or overlapping cohorts 26](#_Toc152749764)

[Table 6 Data Extraction form 26](#_Toc152749764)

Table 1 Search Strategy MEDLINE

| 1 | exp Autoimmune Diseases/ |
| --- | --- |
| 2 | (auto?immune adj2 (disease* or condition*)).mp. |
| 3 | exp Psoriasis/ |
| 4 | psoria$.mp. |
| 5 | exp Vitiligo/ |
| 6 | vitiligo.mp. |
| 7 | (leucoderma or leukoderma).mp. |
| 8 | exp Alopecia Areata/ |
| 9 | alopecia areata.mp. |
| 10 | ((alopecia adj totalis) or (alopecia adj universalis)).mp. |
| 11 | exp Lupus Erythematosus, Systemic/ |
| 12 | (systemic lupus or sle).mp. |
| 13 | arthritis psoriatic.mp. or exp Arthritis, Psoriatic/ |
| 14 | Ankylosing spondylitis.mp. or exp Spondylitis, Ankylosing/ |
| 15 | Spondylarthritis/ |
| 16 | (axial adj2 spondylarthritis).tw. |
| 17 | (axial adj2 spa).tw. |
| 18 | (ankylos$ or spondyl$).tw. |
| 19 | (bekhterev$ or bechterew$).tw. |
| 20 | (Marie adj struempell$).tw. |
| 21 | (AS or axSPA).tw. |
| 22 | Sacroiliitis/ |
| 23 | ((axial or spin$ or peripheral or vertebral or enthesitis) adj3 (joint$ or spondyloarthritis or arthritis or ankylosing)).tw. |
| 24 | exp Arthritis, Rheumatoid/ |
| 25 | ((rheumatoid or reumatoid or revmatoid or rheumatic or reumatic or revmatic or rheumat$ or reumat$ or revmarthrit$) adj3 (arthrit$ or artrit$ or diseas$ or condition$ or nodule$)).mp. |
| 26 | sjogren syndrome.mp. or exp Sjogren's Syndrome/ |
| 27 | exp Celiac Disease/ |
| 28 | (c?eliac adj disease*).mp. |
| 29 | inflammatory bowel disease.mp. or exp Inflammatory Bowel Diseases/ |
| 30 | ulcerative colitis.mp. or exp Colitis, Ulcerative/ |
| 31 | Crohn's disease.mp. or exp Crohn Disease/ |
| 32 | multiple sclerosis.mp. or exp Multiple Sclerosis/ |
| 33 | Systemic Sclerosis.mp. |
| 34 | exp Scleroderma, Systemic/ |
| 35 | ((systemic adj scler?) or sclero?).mp. |
| 36 | crest syndrome.mp. or exp CREST Syndrome/ |
| 37 | graves disease.mp. or exp Graves Disease/ |
| 38 | (grave? adj3 (diseas? or thyrotoxicos? or hyperthyr?)).mp. |
| 39 | exp Thyroiditis, Autoimmune/ |
| 40 | autoimmune thyroid.mp. |
| 41 | Hashimoto Disease.mp. |
| 42 | exp Hashimoto Disease/ |
| 43 | (thyroid adj autoantibodies).mp. [mp=title, book title, abstract, original title, name of substance word, subject heading word, floating sub-heading word, keyword heading word, organism supplementary concept word, protocol supplementary concept word, rare disease supplementary concept word, unique identifier, synonyms] |
| 44 | thyroid.mp. and exp autoantibodies/ |
| 45 | myasthenia gravis.mp. or exp Myasthenia Gravis/ |
| 46 | addison disease.mp. or exp Addison Disease/ |
| 47 | type 1 diabetes.mp. or exp Diabetes Mellitus, Type 1/ or ("typ? 1 diabet*" or "typ? I diabet*" or "typ?1 diabet*" or "typ?I diabet*").mp. or (("insulin* depend*" or "insulin?depend*") not ("non‐insulin* depend*" or "non insulindepend*")).mp. or (IDDM or T1DM or T1D).mp. or autoimmune diabetes.mp. |
| 48 | exp Pregnancy Complications/ |
| 49 | (pregnancy adj complication?).mp. |
| 50 | exp Abortion, Spontaneous/ |
| 51 | ((recurrent adj2 miscarr?) or miscarr?).mp. |
| 52 | (early adj3 pregnancy loss?).mp. |
| 53 | miscarriage.mp. |
| 54 | exp Stillbirth/ |
| 55 | (stillbirth or (still adj birth)).mp. |
| 56 | exp Fetal Death/ |
| 57 | ((f?etal adj death$) or (f?etal adj demise$)).mp. |
| 58 | exp Hypertension, Pregnancy-Induced/ |
| 59 | (gestational hypertension or (pregnancy adj3 hypertensi$)).mp. |
| 60 | (preeclampsia or pre-eclampsia).mp. |
| 61 | exp Pre-Eclampsia/ |
| 62 | (eclampsia or tox?emia).mp. |
| 63 | exp Eclampsia/ |
| 64 | hellp.mp. or exp HELLP Syndrome/ |
| 65 | exp Placenta Accreta/ |
| 66 | (placenta adj accreta).mp. |
| 67 | placenta percreta.mp. |
| 68 | placenta increta.mp. |
| 69 | (morbidly adj3 adherent placenta).mp. |
| 70 | abnormally invasive placenta.mp. |
| 71 | exp Abruptio Placentae/ |
| 72 | Placenta? abruption.mp. |
| 73 | exp Placenta Previa/ |
| 74 | Placenta pr?evia.mp. |
| 75 | low lying placenta.mp. |
| 76 | exp Hyperemesis Gravidarum/ |
| 77 | Hyperemesis Gravidarum.mp. |
| 78 | exp Morning Sickness/ |
| 79 | morning sickness.mp. |
| 80 | exp Diabetes, Gestational/ |
| 81 | GDM.mp. |
| 82 | (((pregnan$ or gestation$ or prenatal$ or antenatal$ or pre-natal$ or ante-natal$ or maternal$) adj2 diabet$) or gestational diabetes).mp. |
| 83 | ((tub$$ adj3 pregnanc$) or (cornual adj3 pregnanc$) or (heterotopic adj3 pregnanc$) or (abdomin$ adj3 pregnanc$) or (extrauterine adj3 pregnanc$) or (interstitial adj3 pregnanc$) or (cervi$ adj3 pregnanc$) or (ovar$ adj3 pregnanc$) or (cesarean scar adj3 pregnanc$)).mp. |
| 84 | exp Pregnancy, Ectopic/ |
| 85 | ectopic pregnancy.mp. |
| 86 | exp Gestational Trophoblastic Disease/ |
| 87 | gestational trophoblastic.mp. |
| 88 | exp Hydatidiform Mole/ |
| 89 | ((hydatid? adj2 mole?) or (molar adj2 pregnanc?)).mp. |
| 90 | exp Choriocarcinoma/ |
| 91 | choriocarcinoma.mp. |
| 92 | exp Pregnancy, Multiple/ |
| 93 | ((pregnanc* or gestation*) adj (twin* or triplet* or quadruplet* or quintuplet* or multiple or multi?f?et*)).mp. |
| 94 | (Monochorionic or dichorionic).mp. |
| 95 | exp Postpartum Hemorrhage/ |
| 96 | (postpartum hemorrhage or post partum hemorrhage or postpartum haemorrhage or post partum haemorrhage).mp. |
| 97 | obstetric haemorrhage.mp. |
| 98 | obstetric labor, premature.mp. |
| 99 | exp Obstetric Labor, Premature/ |
| 100 | (premature labor or premature labour or preterm labor or preterm labour or preterm birth).mp. |
| 101 | exp Cesarean Section/ |
| 102 | exp Cesarean Section, Repeat/ |
| 103 | (caesarean or cesarean or caesarian or cesarian or cesarien or caesarien or c-section or c section).mp. |
| 104 | cesarean.mp. |
| 105 | exp Extraction, Obstetrical/ |
| 106 | exp Obstetrical Forceps/ |
| 107 | ((operative or instrumental or assisted or forcep* or ventouse* or vacuum*) adj1 (deliver* or birth*)).mp. |
| 108 | exp Infant, Low Birth Weight/ |
| 109 | low birth weight.mp. |
| 110 | (low birth weight* adj4 very low birth weight*).mp. |
| 111 | exp Infant, Small for Gestational Age/ |
| 112 | (small adj3 gestational age).mp. |
| 113 | (intra?uterine growth adj2 (restriction* or retardation)).mp. |
| 114 | iugr.ti,ab. |
| 115 | exp Fetal Growth Retardation/ |
| 116 | (fetal growth adj2 (restriction? or retardation)).mp. |
| 117 | exp Depression, Postpartum/ |
| 118 | postpartum depression.mp. |
| 119 | ((postpartum* or post partum* or post-partum* or postnatal* or post natal* or post-natal* or perinatal* or peri natal* or peri-natal* or puerp*) and (depress* or dysthymi* or adjustment disorder* or mood disorder* or affective disorder*)).mp. |
| 120 | ((postpartum* or post partum* or post-partum* or postnatal* or post natal* or post-natal or perinatal* or peri natal* or peri-natal* or puerp*) and (psychos#s or psychotic)).mp. |
| 121 | (psychosis adj3 after childbirth).mp. |
| 122 | (((third or fourth or 3rd or 4th) adj degree) and tear*).mp. |
| 123 | (((anal near adj2 sphincter) or (rectal adj mucosa) or rectum or (anal adj epithelium) or anus or (recto?vaginal adj2 fistulae) or (anorectal adj mucosa) or (anal adj skin)) and (tear* or injur* or damage* or lacerat* or rupture* or trauma)).mp. |
| 124 | ((obstetric* and anal and sphincter and injur*) or (anal and sphincter and injur*)).mp. |
| 125 | (exp Pregnancy/ or exp Obstetrics/ or (pregnan* or obstetric*).mp.) and (exp Cholestasis/ or exp Cholestasis, Intrahepatic/) |
| 126 | pelvic girdle pain.mp. or exp Pelvic Girdle Pain/ |
| 127 | (symphysis pubis adj3 (pain$ or dysfunction$)).mp. |
| 128 | Pubic Symphysis Diastasis.mp. or exp Pubic Symphysis Diastasis/ |
| 129 | Sacroiliac joint dysfunction.mp. |
| 130 | PGP.mp. |
| 131 | pregnancy outcome.mp. or exp Pregnancy Outcome/ |

## Table 2 The Preferred Reporting Items for Systematic reviews and Meta-Analyses (PRISMA)

## Checklist

| **Section and Topic** | | **Item #** | **Checklist item** | | **Location where item is reported** | |  |
| --- | --- | --- | --- | --- | --- | --- | --- |
| **TITLE** | | | | | | |  |
| Title | | 1 | | | Identify the report as a systematic review. | | Page 1 and 2 |
| **ABSTRACT** | | | | | | |  |
| Abstract | | 2 | | | See the PRISMA 2020 for Abstracts checklist. | | Abstract page 1 and 2 |
| **INTRODUCTION** | | | | | | |  |
| Rationale | | 3 | | | Describe the rationale for the review in the context of existing knowledge. | | Background page 2, 3  paragraph 1-2 |
| Objectives | | 4 | | | Provide an explicit statement of the objective(s) or question(s) the review addresses. | | Background page2.3  Paragraph 3 |
| **METHODS** | | | | | | |  |
| Eligibility criteria | | 5 | | | Specify the inclusion and exclusion criteria for the review and how studies were grouped for the syntheses. | | Methods, Page 3,4  paragraph 1 |
| Information sources | | 6 | | | Specify all databases, registers, websites, organisations, reference lists and other sources searched or consulted to identify studies. Specify the date when each source was last searched or consulted. | | Inclusion and exclusion criteria Page 4,5 paragraph 2,3 |
| Search strategy | | 7 | | | Present the full search strategies for all databases, registers and websites, including any filters and limits used. | | Page 3-4 paragraph 1 |
| Selection process | | 8 | | | Specify the methods used to decide whether a study met the inclusion criteria of the review, including how many reviewers screened each record and each report retrieved, whether they worked independently, and if applicable, details of automation tools used in the process. | | Page 3,4 paragraph 1& Appendix Table 1 |
| Data collection process | | 9 | | | Specify the methods used to collect data from reports, including how many reviewers collected data from each report, whether they worked independently, any processes for obtaining or confirming data from study investigators, and if applicable, details of automation tools used in the process. | | Page 3,4  Methods, paragraph 2,3,4 |
| Data items | | 10a | | | List and define all outcomes for which data were sought. Specify whether all results that were compatible with each outcome domain in each study were sought (e.g. for all measures, time points, analyses), and if not, the methods used to decide which results to collect. | | Page 3,4,11,12  Methods, paragraph 2,3,4 |
|  |  | 10b | | | List and define all other variables for which data were sought (e.g. participant and intervention characteristics, funding sources). Describe any assumptions made about any missing or unclear information. | | Page 3,4,12  Methods, paragraph 2,3,4  Table 1-2 |
| Study risk of bias assessment | | 11 | | | Specify the methods used to assess risk of bias in the included studies, including details of the tool(s) used, how many reviewers assessed each study and whether they worked independently, and if applicable, details of automation tools used in the process. | | Page 3  Methods, paragraph 2,3,4  Figure |
| Effect measures | | 12 | | | Specify for each outcome the effect measure(s) (e.g. risk ratio, mean difference) used in the synthesis or presentation of results. | | Page 5-8,22-30  Figure 1  Appendix table 4.1,4.2,4.3 |
| Synthesis methods | | 13a | | | Describe the processes used to decide which studies were eligible for each synthesis (e.g. tabulating the study intervention characteristics and comparing against the planned groups for each synthesis (item #5)). | | Page 3,4  Data analysis |
|  |  | 13b | | | Describe any methods required to prepare the data for presentation or synthesis, such as handling of missing summary statistics, or data conversions. | | Page 3,4  Data analysis |
|  |  | 13c | | | Describe any methods used to tabulate or visually display results of individual studies and syntheses. | | Page 3,4  Methods, paragraph 2,3,4  Table 3 |
|  |  | 13d | | | Describe any methods used to synthesize results and provide a rationale for the choice(s). If meta-analysis was performed, describe the model(s), method(s) to identify the presence and extent of statistical heterogeneity, and software package(s) used. | | Page 3,4  Figure 1  Appendix table 4.1,4.2,4.3 |
|  |  | 13e | | | Describe any methods used to explore possible causes of heterogeneity among study results (e.g. subgroup analysis, meta-regression). | | Page 3,4,5  Data analysis |
|  |  | 13f | | | Describe any sensitivity analyses conducted to assess robustness of the synthesized results. | | Page 4  Data analysis |
| Reporting bias assessment | | 14 | | | Describe any methods used to assess risk of bias due to missing results in a synthesis (arising from reporting biases). | | Page 4  Methods, paragraph 2,3,4  Figure 2 |
| Certainty assessment | | 15 | | | Describe any methods used to assess certainty (or confidence) in the body of evidence for an outcome. | | Page 4,5  Figure 1  Appendix table 4.1,4.2,4.3 |
| **RESULTS** | | | | | | |  |
| Study selection | | 16a | | | Describe the results of the search and selection process, from the number of records identified in the search to the number of studies included in the review, ideally using a flow diagram. | | Page 5-9 |
|  |  | 16b | | | Cite studies that might appear to meet the inclusion criteria, but which were excluded, and explain why they were excluded. | | Appendix table 3 |
| Study characteristics | | 17 | | | Cite each included study and present its characteristics. | | Table 1 |
| Risk of bias in studies | | 18 | | | Present assessments of risk of bias for each included study. | | Figure I and Table 4.1,4.2,4.3 |
| Results of individual studies | | 19 | | | For all outcomes, present, for each study: (a) summary statistics for each group (where appropriate) and (b) an effect estimate and its precision (e.g. confidence/credible interval), ideally using structured tables or plots. | | Results page 5-9 |
| Results of syntheses | | 20a | | | For each synthesis, briefly summarise the characteristics and risk of bias among contributing studies. | | Results page 5-9 |
|  |  | 20b | | | Present results of all statistical syntheses conducted. If meta-analysis was done, present for each the summary estimate and its precision (e.g. confidence/credible interval) and measures of statistical heterogeneity. If comparing groups, describe the direction of the effect. | | Results page 5-9  Figure 1-5 |
|  |  | 20c | | | Present results of all investigations of possible causes of heterogeneity among study results. | | Discussion  Paragraph 2 |
|  |  | 20d | | | Present results of all sensitivity analyses conducted to assess the robustness of the synthesized results. | | NA |
| Reporting biases | | 21 | | | Present assessments of risk of bias due to missing results (arising from reporting biases) for each synthesis assessed. | | NA |
| Certainty of evidence | | 22 | | | Present assessments of certainty (or confidence) in the body of evidence for each outcome assessed. | | NA |
| **DISCUSSION** | | | | | | |  |
| Discussion | | 23a | | | Provide a general interpretation of the results in the context of other evidence. | | Discussion  Page 10-12 |
|  |  | 23b | | | Discuss any limitations of the evidence included in the review. | | Discussion  Page 10-12 |
|  |  | 23c | | | Discuss any limitations of the review processes used. | | Discussion  Page 10-12 |
|  |  | 23d | | | Discuss implications of the results for practice, policy, and future research. | | Discussion  Page 10-12 |
| **OTHER INFORMATION** | | | | | | |  |
| Registration and protocol | | 24a | | | Provide registration information for the review, including register name and registration number, or state that the review was not registered. | | Methods  Page3 |
|  |  | 24b | | | Indicate where the review protocol can be accessed, or state that a protocol was not prepared. | | NA |
|  |  | 24c | | | Describe and explain any amendments to information provided at registration or in the protocol. | | NA |
| Support | | 25 | | | Describe sources of financial or non-financial support for the review, and the role of the funders or sponsors in the review. | | Page 12 |
| Competing interests | | 26 | | | Declare any competing interests of review authors. | | Page 12 |
| Availability of data, code and other materials | | 27 | | | Report which of the following are publicly available and where they can be found: template data collection forms; data extracted from included studies; data used for all analyses; analytic code; any other materials used in the review. | | Page 12 |

## Table 3 List of excluded studies

| Author/year | |  | Reason for exclusion |
| --- | --- | --- | --- |
|  | | Akiyama C, Shirai T, Sato H, Fujii H, Ishii T, Harigae H. Association of various myositis-specific autoantibodies with dermatomyositis and polymyositis triggered by pregnancy. Rheumatology International. 2022;42(7):1271-80. | Outcome |
|  | | Akkaya C, Kocagoz SZ, Turan OF, Taskapilioglu O, Kirli S. Onset of multiple sclerosis following post-partum depressive and manic episodes. Psychiatry and Clinical Neurosciences. 2007;61(6):698-9. | Study design  Letter to editor |
|  | | Alijotas-Reig J, Esteve-Valverde E. Pregnancy and autoimmune diseases. Medicina Clinica. 2017;148(4):161-3. | Study design |
|  | Auger N, Quach C, Healy-Profitos J, Dinh T, Chasse M. Early predictors of Guillain-Barre syndrome in the life course of women. International Journal of Epidemiology. 2018;47(1):280-8. | | Outcome |
|  | Badihian N, Riahi R, Goli P, Badihian S, Poursafa P, Kelishadi R. Prenatal and perinatal factors associated with developing multiple sclerosis later in life: A systematic review and meta-analysis. Autoimmunity Reviews. 2021;20(6):102823. | | Population |
|  | Barrett PM, McCarthy FP, Evans M, Kublickas M, Perry IJ, Stenvinkel P, et al. Stillbirth is associated with increased risk of long-term maternal renal disease: a nationwide cohort study. American Journal of Obstetrics & Gynecology. 2020;223(3):427.e1-.e14. | | Outcome |
|  | | Boldingh MI, Maniaol AH, Brunborg C, Weedon-Fekjaer H, Verschuuren JJ, Tallaksen CM. Increased risk for clinical onset of myasthenia gravis during the postpartum period. Neurology. 2016;87(20):2139-45. | Exposure |
|  | Brown HK, Barrett K, Scime NV. Perinatal mental illness and maternal autoimmune disease: A review of current evidence and avenues for future research. Frontiers in Neuroendocrinology. 2022;65:100975. | | Study design |
|  | Dhillon-Smith RK, Tobias A, Smith PP, Middleton LJ, Sunner KK, Baker K, et al. The Prevalence of Thyroid Dysfunction and Autoimmunity in Women With History of Miscarriage or Subfertility. Journal of Clinical Endocrinology & Metabolism. 2020;105(8):01. | | Outcome |
|  | | Dissanayake TD, Maksymowych WP, Keeling SO. Peripartum issues in the inflammatory arthritis patient: A survey of the RAPPORT registry. Scientific Reports. 2020;10(1):3733. | Comparator group not as per inclusion criteria |
|  | Driul L, Bertozzi S, Londero AP, Fruscalzo A, Rusalen A, Marchesoni D, et al. Risk factors for chronic pelvic pain in a cohort of primipara and secondipara at one year after delivery: association of chronic pelvic pain with autoimmune pathologies. Minerva Ginecologica. 2011;63(2):181-7. | | Outcome |
|  | | Efe C, Purnak T, Ozaslan E. Autoimmune hepatitis in the postpartum period. Clinics & Research in Hepatology & Gastroenterology. 2012;36(4):391-3. | Study design |
|  | | Erlinger S. Intrahepatic cholestasis of pregnancy: A risk factor for cancer, autoimmune and cardiovascular diseases? Clinics & Research in Hepatology & Gastroenterology. 2016;40(2):139-40. | Study design |
|  | Gibbins KJ, Tebo AE, Nielsen SK, Branch DW. Antiphospholipid antibodies in women with severe preeclampsia and placental insufficiency: a case-control study. Lupus. 2018;27(12):1903-10. | | Outcome |
|  | | Gleicher N. Postpartum depression, an autoimmune disease? Autoimmunity Reviews. 2007;6(8):572-6. | Outcome |
|  | | Gokce A, Benlioglu C, Baydemir K, Kalafat E, Atabekoglu C. Cesarean delivery: A predisposing factor for autoimmune thyroid disease in iodine replete women? European Journal of Obstetrics, Gynecology, & Reproductive Biology. 2018;224:131-2. | Study design |
|  | | Gouveri E, Tews D. A case of type 1 diabetes mellitus after gestational diabetes. Hippokratia. 2021;25(2):95. | Study design |
|  | Honig A, Engel JB, Segerer SE, Kranke P, Hausler S, Wurfel W. Pregnancy-triggered antiphospholipid syndrome in a patient with multiple late miscarriages. Human Reproduction. 2010;25(11):2753-4. | | Outcome |
|  | | Incani M, Baroni MG, Cossu E. Testing for type 1 diabetes autoantibodies in gestational diabetes mellitus (GDM): is it clinically useful? BMC Endocrine Disorders. 2019;19(1):44. | Study design |
|  | | Jorgensen KT, Pedersen BV, Nielsen NM, Jacobsen S, Frisch M. Childbirths and risk of female predominant and other autoimmune diseases in a population-based Danish cohort. Journal of Autoimmunity. 2012;38(2-3):J81-7. | Exposure |
|  | | Kalra B, Kalra S, Chhabra B. Posthumous Caesarean section in women with type 1 diabetes mellitus: two cases at one hospital in Northern India. Diabetologia. 2010;53(11):2452-3. | Study design |
|  | | Kirty K, Sarda Y, Jacob A, Venugopala D. Wernicke's encephalopathy post hyperemesis gravidarum misdiagnosed as Guillain-Barre syndrome: lessons for the frontline. BMJ Case Reports. 2021;14(3):16. | Study design |
|  | | Kiss E, Kiss CG, Poor G. [Systemic autoimmune disorders and pregnancy]. Orvosi Hetilap. 2011;152(43):1715-23. | Outcome |
|  | | Kitzmiller JL, Ferrara A, Peng T, Cissell MA, Kim C. Preexisting Diabetes and Pregnancy. National Institute of Diabetes and Digestive and Kidney Diseases. 2018;5:08. | Outcome |
|  | | Kopp TI, Pinborg A, Glazer CH, Magyari M. Women with female infertility seeking medically assisted reproduction are not at increased risk of developing multiple sclerosis. Human Reproduction. 2022;37(6):1324-33. | Population |
|  | Koslowsky B, Grisaru-Granovsky S, Livovsky DM, Milgrom Y, Goldin E, Bar-Gil Shitrit A. Pregnancy-Onset Inflammatory Bowel Disease: A Subtle Diagnosis. Inflammatory Bowel Diseases. 2018;24(8):1826-32. | | Population |
|  | | Kotsev S. Ogilvie's syndrome following cesarean delivery: The Dubai's case. Saudi Journal of Anaesthesia. 2011;5(3):335-8. | Study design and Outcome |
|  | | Koubi M, Rossi P, Arcani R, Gomes De Pihno Q, Chau C, Blanc J, et al. Relevance of systematic anti-nuclear antibodies testing after obstetrical complications. Journal of Reproductive Immunology. 2021;148:103437. | Outcome |
|  | | Lewandowski K, Hincz P, Grzesiak M, Cajdler-Luba A, Salata I, Wilczynski J, et al. New onset Addison's disease presenting as prolonged hyperemesis in early pregnancy. Ginekologia Polska. 2010;81(7):537-40. | Study design |
|  | Li X, Zhang W, Lin J, Liu H, Yang Z, Teng Y, et al. Risk factors for adverse maternal and perinatal outcomes in women with preeclampsia: analysis of 1396 cases. Journal of Clinical Hypertension. 2018;20(6):1049-57. | | outcome |
|  | Liu B, Song L, Li H, Zheng X, Yuan J, Liang Y, et al. History of spontaneous miscarriage and the risk of diabetes mellitus among middle-aged and older Chinese women. Acta Diabetologica. 2018;55(6):579-84. | | Outcome-add to discussion |
|  | McDonald SD, Han Z, Walsh MW, Gerstein HC, Devereaux PJ. Kidney disease after preeclampsia: a systematic review and meta-analysis. American Journal of Kidney Diseases. 2010;55(6):1026-39. | | Outcome |
|  | McHugh NJ, Reilly PA, McHUGH LA. Pregnancy outcome and autoantibodies in connective tissue disease. The Journal of rheumatology. 1989;16(1):42-6. | | Comparator |
|  | | Mittal A, Dexter S, Marcus S, Tremble J. First presentation of Addison's disease in the 2nd trimester of pregnancy. Journal of Obstetrics & Gynaecology. 2011;31(4):342. | Study design |
|  | Moberg JY, Laursen B, Thygesen LC, Magyari M. Reproductive history of the Danish multiple sclerosis population: A register-based study. Multiple Sclerosis. 2020;26(8):902-11. | | Outcome |
|  | Munoz CM, Goulden B, Ahmed K, Alijotas-Reig J, Giles I. Risk of adverse pregnancy outcomes prior to the onset of an autoimmune rheumatic disease: a systematic review. Rheumatology. 2022;05:05. | | Study design |
|  | | Negishi M, Shimomura K, Proks P, Nakahara R, Murakami M, Shimomura Y, et al. Development of postpartum Graves’ disease and type 1 diabetes after delivery in a patient with gestational diabetes. Journal of Diabetes Investigation. 2011;2(4):328-30. | Study design |
|  | Nguyen AL, Vodehnalova K, Kalincik T, Signori A, Havrdova EK, Lechner-Scott J, et al. Association of Pregnancy With the Onset of Clinically Isolated Syndrome. JAMA Neurology. 2020;77(12):1496-503. | | Population |
|  | Nicolini G, Degli Esposti D, Ianniello E, Moroni L, Bacchelli S, Cicero AFG, et al. Long-term consequences of previous preeclampsia and complicated pregnancy: analysis of echocardiographic aspects. Journal of Cardiovascular Medicine. 2021;22(12):939-45. | | outcome |
|  | | O'Donoghue K. Pregnancy and the risk of autoimmune disease: An exploration. Chimerism. 2011;2(3):84-5. | Outcome |
|  | | Okoth K, Subramanian A, Chandan JS, Adderley NJ, Thomas GN, Nirantharakumar K, et al. Long term miscarriage-related hypertension and diabetes mellitus. Evidence from a United Kingdom population-based cohort study. PLoS ONE [Electronic Resource]. 2022;17(1):e0261769. | Outcome |
|  | | Ostensen M, Villiger PM, Forger F. Interaction of pregnancy and autoimmune rheumatic disease. Autoimmunity Reviews. 2012;11(6-7):A437-46. | Outcome |
|  | | Perricone C, de Carolis C, Perricone R. Pregnancy and autoimmunity: a common problem. Best Practice & Research in Clinical Rheumatology. 2012;26(1):47-60. | Study design |
|  | Peschken CA, Robinson DB, Hitchon CA, Smolik I, Hart D, Bernstein CN, et al. Pregnancy and the risk of rheumatoid arthritis in a highly predisposed North American Native population. Journal of Rheumatology. 2012;39(12):2253-60. | | Population |
|  | | Petri M, Allbritton J. Fetal outcome of lupus pregnancy: a retrospective case-control study of the Hopkins Lupus Cohort. Obstetrical & gynecological survey. 1993;48(11):717-8. | Case series |
|  | Rajewski M, Skrzypczak J. [Frequency of antiphospholipid antibodies and antiphospholipid syndrome in women with recurrent miscarriages]. Ginekologia Polska. 2011;82(1):32-8. | | Outcome |
|  | Silman AJ, Roman E, Beral V, Brown A. Adverse reproductive outcomes in women who subsequently develop rheumatoid arthritis. Ann Rheum Dis. 1988;47(12):979-81. | | Comparator |
|  | Tabacco S, Ludovisi M, D'Alfonso A, Palermo P, Roncone R, T DEV, et al. Perinatal mental illness and autoimmune diseases: a qualitative sistematic review. Minerva Obstetrics and Gynecology. 2022;02:02. | | Study design |
|  | | Ting Tai Y, Mohd Noor N. A rare case of a mother with gestational diabetes complicated with fulminant type 1 diabetes mellitus post-delivery. Journal of the Royal College of Physicians of Edinburgh. 2022;52(2):120-3. | Study design |
|  | Tufano A, Coppola A, Maruotti GM, Martinelli P, Cerbone AM, Di Minno G. HELLP syndrome and its relation with the antiphospholipid syndrome. Blood Transfusion. 2014;12(1):114-8. | | Outcome |
|  | | Unnikrishnan R, Shanthi Rani CS, Anjana RM, Uthra SC, Vidya J, Sankari GU, et al. Postpartum development of type 1 diabetes in Asian Indian women with gestational diabetes. Indian Journal of Endocrinology and Metabolism. 2016;20(5):702-6. | Study design |
|  | van den Boogaard E, Cohn DM, Korevaar JC, Dawood F, Vissenberg R, Middeldorp S, et al. Number and sequence of preceding miscarriages and maternal age for the prediction of antiphospholipid syndrome in women with recurrent miscarriage. Fertility & Sterility. 2013;99(1):188-92. | | Outcome |
|  | Wucher H, Lepercq J, Timsit J. Onset of autoimmune type 1 diabetes during pregnancy: Prevalence and outcomes. Best Practice & Research Clinical Endocrinology & Metabolism. 2010;24(4):617-24. | | Study design |
|  | You Q, Jiang Q, Shani I, Lou Y, Huang S, Wang S, et al. Miscarriage, stillbirth and the risk of diabetes in women: A systematic review and meta-analysis. Diabetes Research and Clinical Practice. 2023;195:110224. | | Outcome |
|  | | Zhang L, Ding D, Yu L, Qi H, Han C, Jiang J, et al. Primary biliary cirrhosis associated with myasthenia gravis after postpartum: a case report. Journal of Medical Case Reports [Electronic Resource]. 2021;15(1):498. | Study design |

## Table 4.1 Quality assessment of the Cohort studies using NOS (Newcastle Ottawa scale)

| Author  Year | Representativeness of the cohort | Selection of the non exposed cohort | Ascertainment of Exposure | Demonstration that outcome of interest was not present at start of stud | Comparability of cohorts on the basis of the design or analysis | Assessment of AI | Was follow-up long enough | Adequacy of follow up | Overall Score | Sample size |
| --- | --- | --- | --- | --- | --- | --- | --- | --- | --- | --- |
| Auvinen 2020 | Low | High | Low | Unclear | Low | High | Low | Low | Fair | 782 |
| Bergink 2011 | Low | Low | Low | Low | Unclear | Low | Low | High | Good | 148 |
| Bergink 2018 | Low | Low | Low | Low | Unclear | Low | Low | High | Fair | 312779 |
| Brann  2023 | Low | Low | Low | Low | Unclear | Low | Low | High | Fair | 530,397 |
| Harpsoe 2013 | Low | Low | Low | Low | Low | Low | Low | Low | Good | 55699 |
| Jorgensen KT 2014 Increased | Low | Unclear | Low | Low | Low | Low | Low | Low | Good | 97077 |
| Jørgensen KT 2012 hyper | Low | Low | Low | Unclear | Low | Low | Low | Low | Good | 1564567 |
| Jørgensen KT 2010 national | Unclear | Low | Low | Low | Low | Low | Low | Unclear | Fair | 2140056 |
| Kamper 2018 | Low | Low | Low | Unclear | Unclear | Low | Low | Low | Good | 1537747 |
| Khashan 2011 | Low | Low | Low | Low | Unclear | Low | Low | Low | Good | 1035639 |
| Lee 2022 | Low | Low | Low | Low | High | Low | Low | Unclear | Fair | 2260952 |
| Lin 2016 | Low | Low | Low | Low | Low | Low | Low | Unclear | Good | 58421 |
| Lin 2018 | Low | Low | Low | Low | High | Low | Low | Low | Fair | 145455 |
| Mao 2022 | Low | Low | Low | Unclear | Low | Low | Low | Low | Good | 11997 |
| Mikkelsen 2022 | Low | Low | Low | Unclear | Low | Low | Low | Low | Good | 1513544 |
| Nielsen 2011 | Low | Low | Low | Low | Unclear | Low | Low | Low | Good | 2140000 |
| Savitz 2014 | Unclear | Low | Low | Low | Low | Low | Low | High | Fair | 978545 |
| Stuart 2018 | Low | Low | Low | Low | Low | Low | Low | Unclear | Good | 1873440 |
| Ulff-Møller 2009 | Low | Low | Low | Low | Unclear | Low | Low | Low | Good | 2140000 |
| Wikstrom 2015 | Low | Low | Low | Low | Unclear | Low | Low | Low | Good | 125281 |

## Table 4.2 Quality assessment of the case control studies using NOS (Newcastle Ottawa scale)

| Authors | Is the case definition adequate | Representativeness of the cases | Selection of Controls | Definition of Controls | Comparability of cases and controls on the basis of the design or analysis | Ascertainment of exposure | Same method of ascertainment for cases and controls | Non-Response rate | Overall score | Sample size |
| --- | --- | --- | --- | --- | --- | --- | --- | --- | --- | --- |
| Hardy 1999 | Low | Low | Low | Unclear | Low | Low | Low | High | Fair | 414 |
| Julkunen 1993 | Low | high | Low | Unclear | Low | Low | Low | High | Fair | 898 |
| Kay 1965 | Low | Unclear | Low | Low | Low | Low | Low | low | Good | 418 |
| Kither 2020 | Low | Low | Low | Low | Low | Low | Low | Unclear | Good | 117446 |
| Ma 2014 | Low | Unclear | Unclear | Low | Low | Low | Low | Unclear | Good | 1102 |
| Siamopoulou 1988 | Low | Low | Unclear | Low | Low | Low | Low | Unclear | Good | 419 |
| Spector 1990 | Low | Low | Low | Low | Low | Unclear | Low | Low | Good | 657 |
| Van Wyk 2011 | Low | Low | Unclear | Low | Low | Low | Low | Low | Good | 206 |
| Wallenius 2011 | Low | Low | Low | Low | Low | Low | Low | Unclear | Good | 2200000 |

## Table 4.3 Quality assessment of the cross-sectionall studies using NOS (Newcastle Ottawa scale)

| Authors/year | Representativeness of the sample | Sample size | Non respondants | Ascertainment of the exposure (risk factor) | Comparability of subjects in different outcome groups on the basis of design or analysis. Confounding factors controlled | Assessment of outcome: | Statistical test: | Overall | Weight |
| --- | --- | --- | --- | --- | --- | --- | --- | --- | --- |
| Hee 2022 | Low | Low | Unclear | Low | Low | Low | Low | Good | 299629 |

__________________________________________________________________________

## Figure 1.1 Meta-analysis of two studies reporting association of miscarriage and future development of SLE


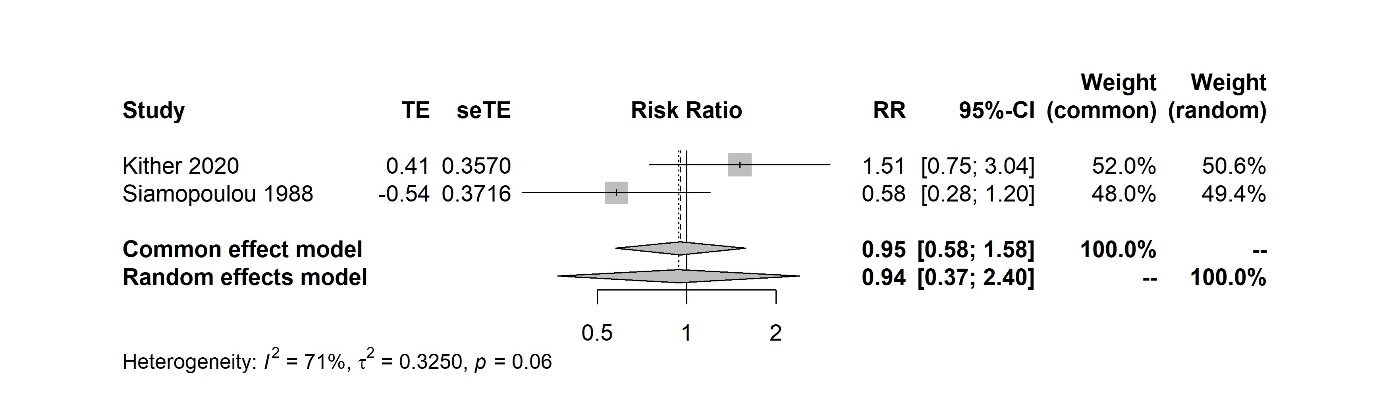


## Figure 1.2 Meta-analysis of two studies reporting association of miscarriage and future development of rheumatoid arthritis (odds ratios)


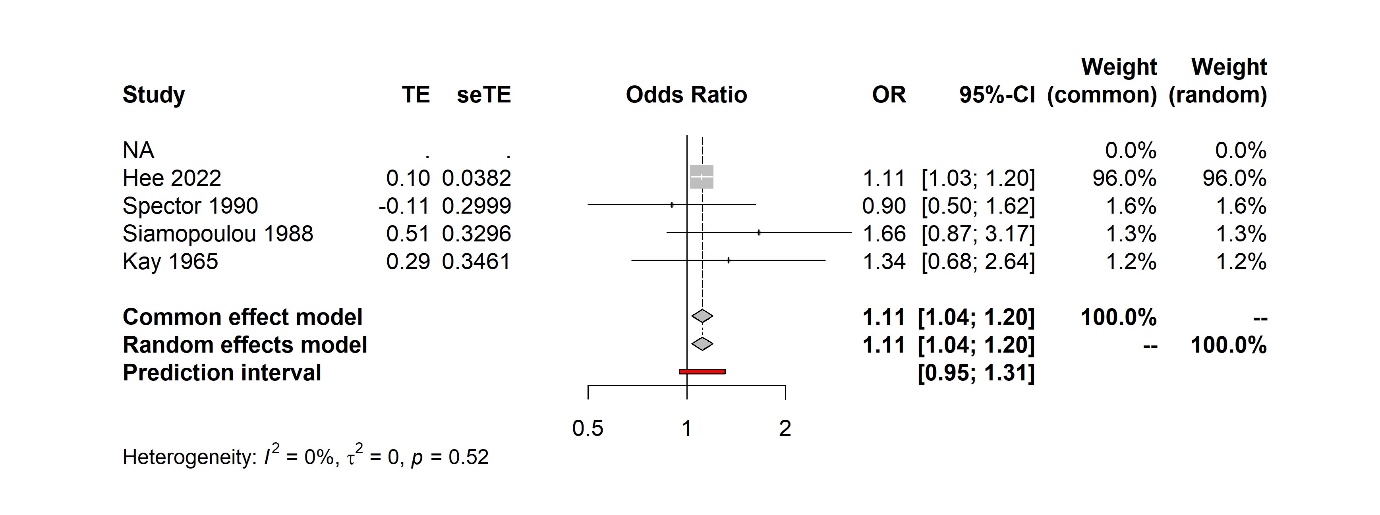


## Figure 1.3 Meta-analysis of two studies reporting association of induced abortion and future development of rheumatoid arthritis


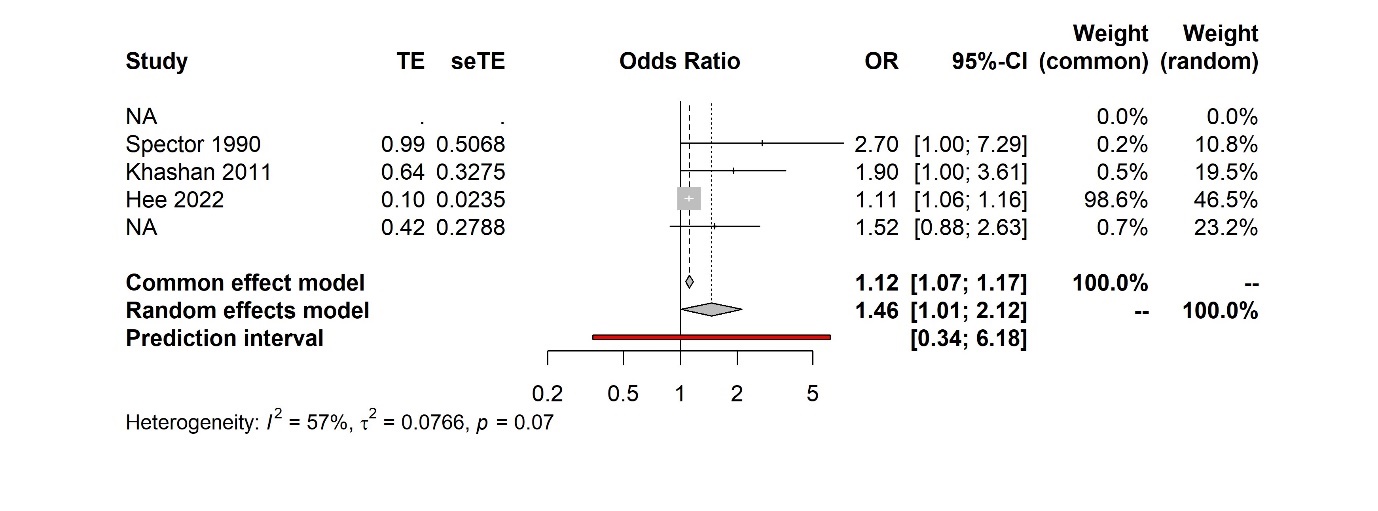


## Figure 1.4 Meta-analysis of two studies reporting association of stillbirth and future development of SLE(risk ratio)


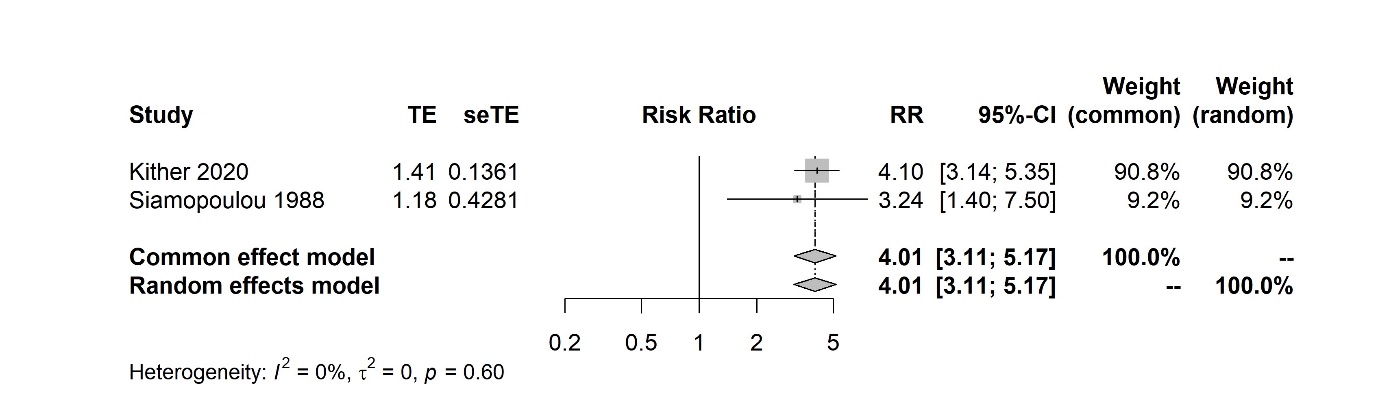


## Figure 1.5 Meta-analysis of two studies reporting association of stillbirth and future development of rheumatoid arthritis


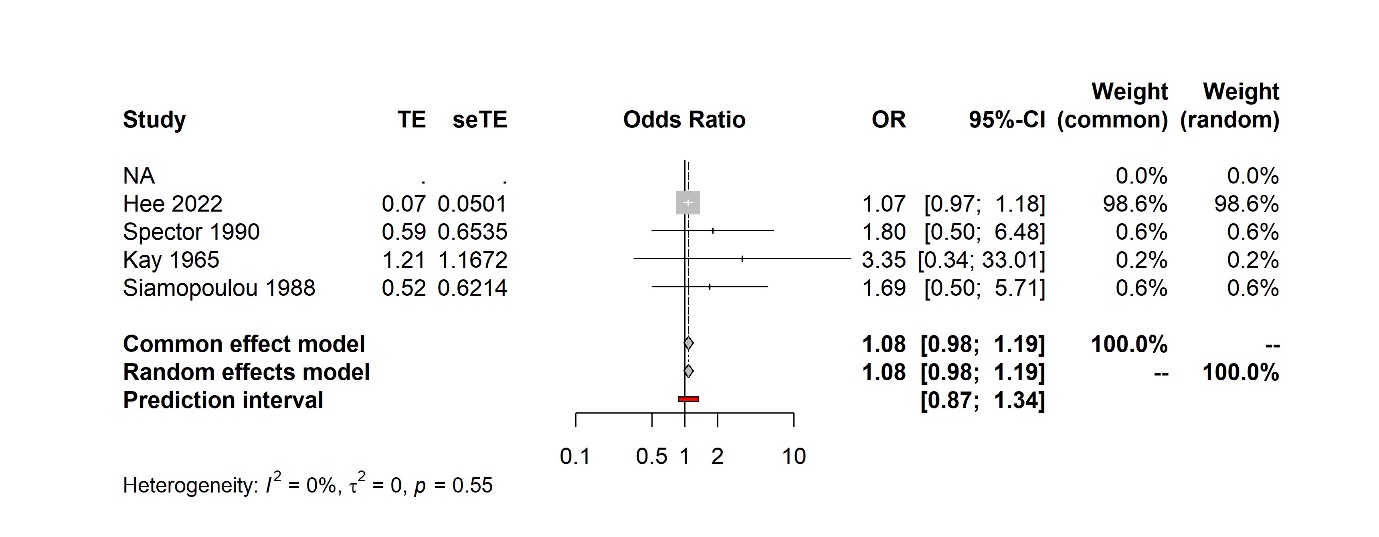


## Figure 1.6 Meta-analysis of two studies reporting association of gestational hypertension or pre-eclampsia and future development of SLE


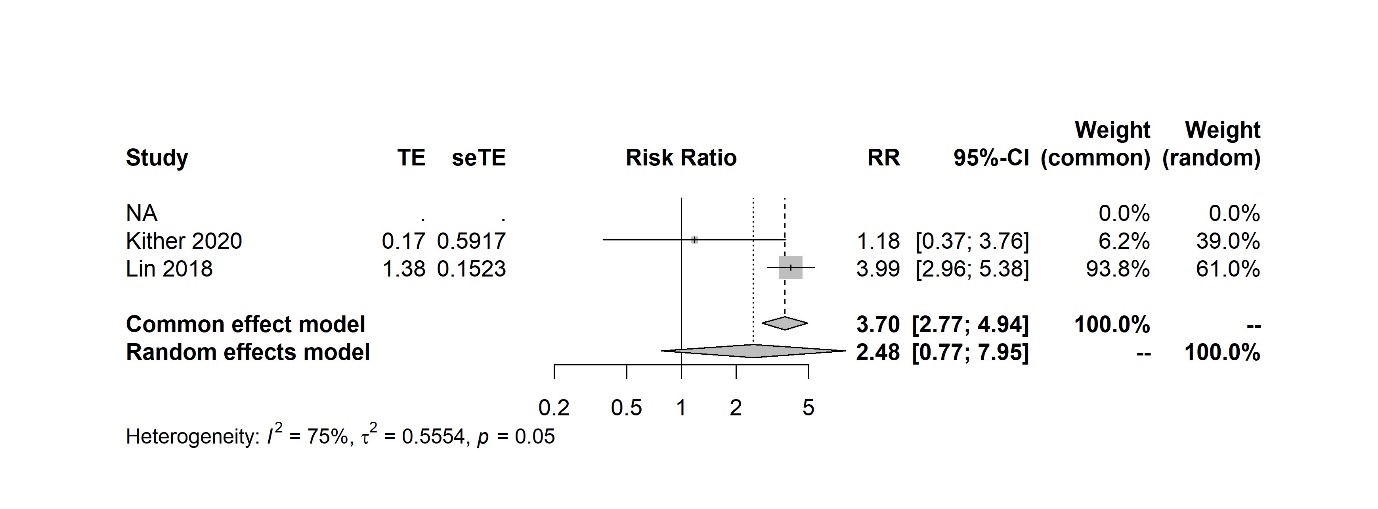


## Figure 1.7 Meta-analysis of two studies reporting association of preterm birth and future development of SLE


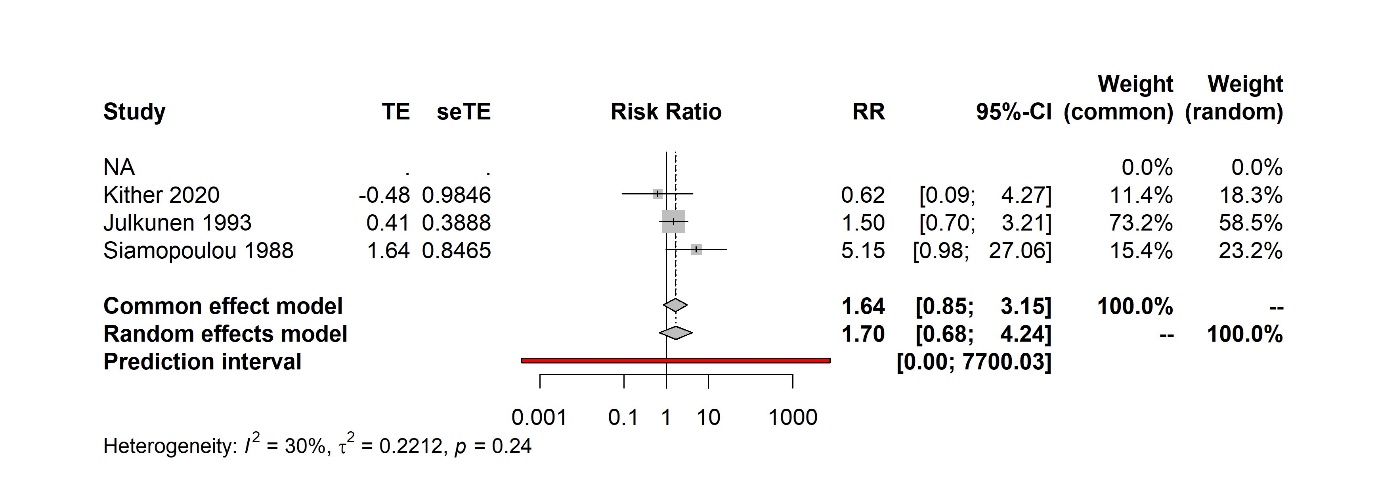


## Figure 1.8 Meta-analysis of two studies reporting association of preterm birth and future development of rheumatoid arthritis


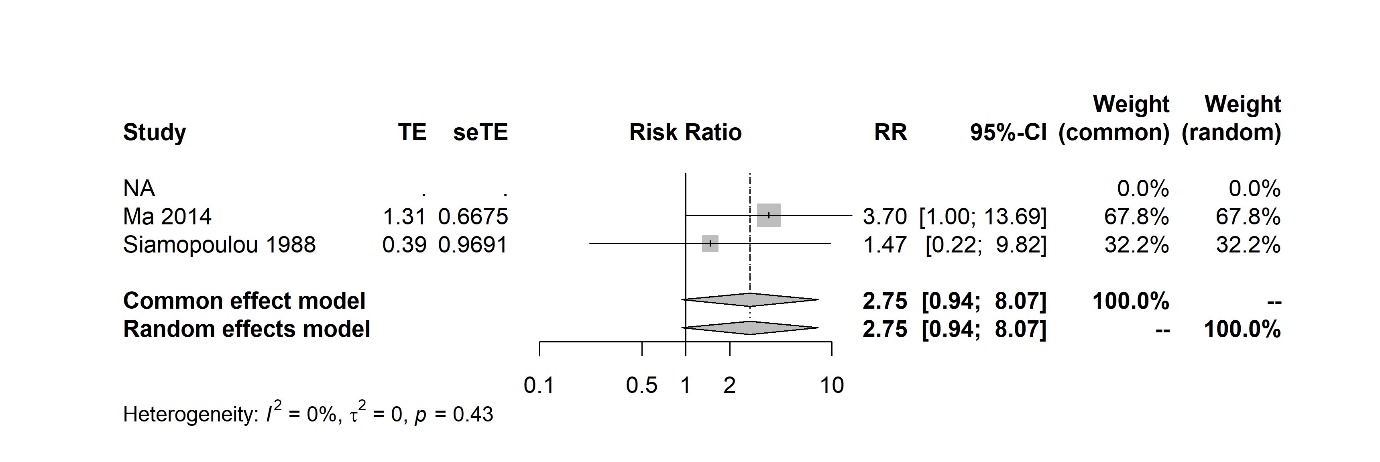


## Figure 1.9 Meta-analysis of two studies reporting association of gestational diabetes and future development of T1DM


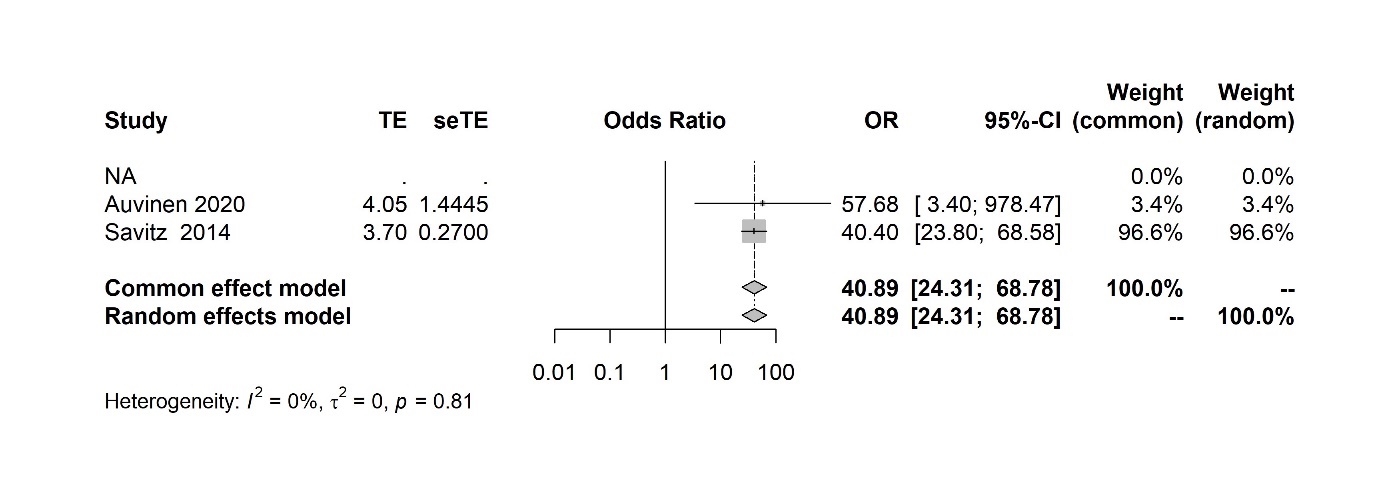


##

## Figure 1.10 The new findings from this review


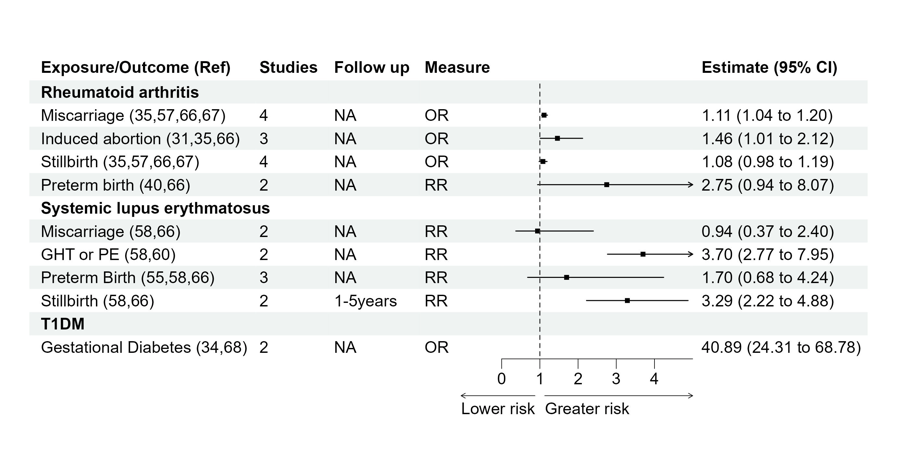


GHT or PE=Gestational hypertension or pre-eclampsia, , HR=hazard ratio, RR=risk ratio, IRR=incidence rate ratio, OR=odds ratio, NA=not available, ‘*’=Case control study

## Figure 1.11 The mixed findings of this review


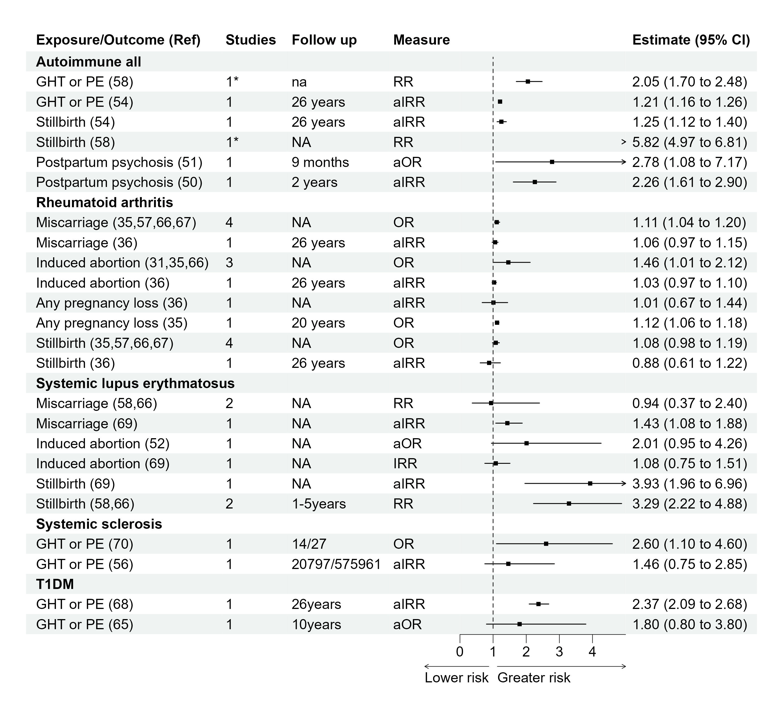


GHT or PE=Gestational hypertension or pre-eclampsia, , HR=hazard ratio, RR=risk ratio, IRR=incidence rate ratio, OR=odds ratio, NA=not available, ‘*’=Case control study

## Table 5 Cohort studies with same or overlapping cohorts

| Study | Cohort (all cohort studies) | Exposure | Outcome |
| --- | --- | --- | --- |
| 1. Ulff-Møller,2009 | 1935-1989  The cohort consisted of 4.4 million women and men born between 1935 and 1989. | Abortion (spontaneous, missed, induced),  ectopic pregnancy, molar pregnancy | Systemic lupus erthematosus |
| 1. Jorgensen, 2010   Extended report | 1935-1989  women and men born in 1935–89. This cohort was linked to national health registries | Abortion (spontaneous, missed, induced),  ectopic pregnancies,  hydatidiform moles,  hyperemesis gravidarum, gestational hypertension, and pre-eclampsia | Rheumatoid arthritis |
| 1. Neilson 2011 | 1935-1989  all Danish women and men (4.4 million) born between 1935 and 1989 and alive in 1968 or later | Miscarriage, stillbirth  ectopic pregnancy,  hyperemesis gravidarum, gestational hypertension, pre-eclampsia) | Multiple sclerosis |
| 1. Khashan, 2011 | 1962-1992  all women born in Denmark between January 1, 1962 and December 31, 1992. | Induced abortion, Caesarean section | All autoimmune diseases, rheumatoid arthritis, multiple sclerosis, inflammatory bowel disease |
| 1. Jorgensen, 2012 | 1955-1993  Danish women born between 1955 and 1993 | Abortion (spontaneous, missed, induced),  ectopic pregnancies  Stillbirth  Hyperemesis gravidarum, gestational hypertension, preeclampsia, | Various autoimmune diseases |
| 1. Harpose 2013 | Pregnancies 1996-2002  completing 86,054 full-term pregnancies (.37 weeks of gestation). | Gestational hypertension, pre-eclampsia, hyperemesis gravidarum | Inflammatory bowel disease |
| 1. Jorgensen, 2014 | Pregnancies 1996-2002 | Gestational hypertension,  pre-eclampsia, hyperemesis gravidarum | Rheumatoid arthritis |
| 1. Kamper 2018 | Women born 1978-2010 | Pre-eclampsia | Systemic sclerosis |
| 1. Mikkelsen,2022 | 1957-1977  All women born between 1957–1997 and living in Denmark between 1977–2017 | Miscarriage | Myasthenia gravis |

## Table 6 Data Extraction form

| Author/Year |
| --- |
| Geographical area |
| Population |
| Database/heath care setting |
| Exposure |
| Comparator |
| Outcomes |
| Definition of exposure and ascertainment methods |
| Definition of outcome and ascertainment methods |
| Study design |
| Age mean control |
| Age mean case |
| Data analysis method |
| Population case |
| Comparator case |
| Population control |
| Comparator control |
| Total number |
| Summary estimate |
| i2 |
| Follow up period |
| Author's conclusion |
| Strengths and limitations of the study |
| Funding |
